# Genome-wide analyses of human noroviruses provide insights on evolutionary dynamics and evidence of coexisting viral populations evolving under recombination constraints

**DOI:** 10.1371/journal.ppat.1009744

**Published:** 2021-07-13

**Authors:** Kentaro Tohma, Cara J. Lepore, Magaly Martinez, Juan I. Degiuseppe, Pattara Khamrin, Mayuko Saito, Holger Mayta, Amy U. Amanda Nwaba, Lauren A. Ford-Siltz, Kim Y. Green, Maria E. Galeano, Mirko Zimic, Juan A. Stupka, Robert H. Gilman, Niwat Maneekarn, Hiroshi Ushijima, Gabriel I. Parra

**Affiliations:** 1 Division of Viral Products, CBER, FDA, Silver Spring, Maryland, United States of America; 2 IICS, National University of Asuncion, Asuncion, Paraguay; 3 INEI-ANLIS, Ministry of Health, Buenos Aires, Argentina; 4 Department of Microbiology, Faculty of Medicine, Chiang Mai University, Chiang Mai, Thailand; 5 Department of Virology, Tohoku University Graduate School of Medicine, Sendai, Japan; 6 Department of Cellular and Molecular Sciences, Faculty of Sciences, Universidad Peruana Cayetano Heredia, Lima, Peru; 7 Laboratory of Infectious Diseases, NIAID, NIH, Bethesda, Maryland, United States of America; 8 Department of International Health, Johns Hopkins University Bloomberg School of Public Health, Baltimore, Maryland, United States of America; 9 Division of Microbiology, Department of Pathology and Microbiology, Nihon University School of Medicine, Tokyo, Japan; Wenzhou-Kean University, CHINA

## Abstract

Norovirus is a major cause of acute gastroenteritis worldwide. Over 30 different genotypes, mostly from genogroup I (GI) and II (GII), have been shown to infect humans. Despite three decades of genome sequencing, our understanding of the role of genomic diversification across continents and time is incomplete. To close the spatiotemporal gap of genomic information of human noroviruses, we conducted a large-scale genome-wide analyses that included the nearly full-length sequencing of 281 archival viruses circulating since the 1970s in over 10 countries from four continents, with a major emphasis on norovirus genotypes that are currently underrepresented in public genome databases. We provided new genome information for 24 distinct genotypes, including the oldest genome information from 12 norovirus genotypes. Analyses of this new genomic information, together with those publicly available, showed that (i) noroviruses evolve at similar rates across genomic regions and genotypes; (ii) emerging viruses evolved from transiently-circulating intermediate viruses; (iii) diversifying selection on the VP1 protein was recorded in genotypes with multiple variants; (iv) non-structural proteins showed a similar branching on their phylogenetic trees; and (v) contrary to the current understanding, there are restrictions on the ability to recombine different genomic regions, which results in co-circulating populations of viruses evolving independently in human communities. This study provides a comprehensive genetic analysis of diverse norovirus genotypes and the role of non-structural proteins on viral diversification, shedding new light on the mechanisms of norovirus evolution and transmission.

## Author summary

Norovirus is a highly diverse enteric pathogen. The large genomic database accumulated in the last three decades advanced our understanding of norovirus diversity; however, this information is limited by geographical bias, sporadic times of collection, and missing or incomplete genome sequences. In this multinational collaborative study, we mined archival samples collected since the 1970s and sequenced nearly full-length new genomes from 281 historical noroviruses, including the first full-length genomic sequences for three genotypes. Using this novel dataset, we found evidence for restrictions in the recombination of genetically disparate viruses and that diversifying selection results in new variants with different epidemiological profiles. These new insights on the diversification of noroviruses could provide baseline information for the study of future epidemics and ultimately the prevention of norovirus infections.

## Introduction

Norovirus is the major cause of acute gastroenteritis in all age groups. The most common symptoms of norovirus infection are diarrhea, vomiting, nausea, and abdominal cramps. In healthy individuals the disease is resolved rapidly, but in vulnerable populations (e.g. the elderly, malnourished children, or immunocompromised patients) symptoms can last longer, resulting in severe dehydration and death. Annually, norovirus is associated with over 600 million cases of acute gastroenteritis and up to 200,000 deaths in children, mostly from developing countries [1,2]. Thus, a vaccine could save hundreds of thousands of lives and help to mitigate the burden of norovirus disease.

The human norovirus genome is a single-stranded, positive-sense, polyadenylated, RNA molecule of ~7.5kb in size, organized into three open reading frames (ORFs) flanked by two short untranslated regions. ORF1 encodes the non-structural proteins (NS1/2-7), required for virus replication, while ORF2 and ORF3 encode the major (VP1) and minor (VP2) capsid proteins, respectively [3]. The genome is enclosed by an array of capsid proteins that form icosahedral particles with a diameter of approximately 27 nm [4]. The structural model of norovirus VP1 presents two domains, the shell (S) and the protruding (P) domain [5]. The S domain forms the scaffold of the icosahedral capsid, while the P domain is a projection that contains the determinants of host interactions, including neutralizing epitopes and carbohydrate binding sites that act as attachment factors to facilitate infection [6–8]. While the precise role of VP2 remains to be elucidated, there is evidence that it might be involved in particle stabilization [9].

One of the major obstacles for norovirus vaccine development is the large viral genetic and antigenic diversity [10]. Genetic differences within VP1 have been used to classify noroviruses into genogroups and genotypes [11]. Over 30 different genotypes, mostly from genogroups I (GI) and II (GII), have been shown to infect humans. While most genotypes circulate with variable incidences, a single genotype (GII.4) predominates globally [12,13]. This dominance of GII.4 viruses has been explained by the accrual of mutations on the VP1, resulting in the emergence of new variants [14–16]. Although GII.4 predominance has been recorded for over two decades, other genotypes can transiently predominate in a given geographical location. This is evidenced by the recent emergence and increase in incidence of GII.17 and GII.2 in different countries [17–21]. While these two viruses are antigenically distinct to GII.4 [8,22], suggesting immune escape as a mechanism of emergence, it seems that norovirus emergence is a multifactorial event that includes changes in viral proteins other than VP1 and virus-host interactions [12]. Thus, the emergence of epidemic GII.2 viruses was associated with changes in the NS7 (viral RNA polymerase) [23], and the emergence of epidemic GII.17 viruses was associated with mutations in VP1 that resulted in the emergence of a new variant and changes in host susceptibility [24–26].

It has been shown that noroviruses are prone to recombine at the ORF1/ORF2 boundaries [27]. Thus, because norovirus also presents different types of viral RNA polymerases [11], viruses with different combinations of capsid and polymerase types have been detected in nature [27–29]. While most recombinant viruses have been found circulating at low levels in the human population, emergence of epidemic viruses has also been associated with recombination events. The most recent example is the emergence of different viruses associated with the GII.P16 polymerase type causing outbreaks around the world [30]. It seems that mutations in this viral polymerase provided new characteristics for successful human infection, as shown by the large outbreaks of GII.2[P16] (GII.2 capsid and GII.P16 polymerase) viruses reported in the 2016/2017 season. This polymerase was initially associated with GII.2 and GII.4 capsids [17,31], but recently this polymerase has been reported with multiple other capsids, including GII.1, GII.3, GII.12, and GII.13 [30,32]. The mechanism by which the viral polymerase is involved in viral emergence is not completely understood, but might include differences in the mutational and/or transcriptional rate that could facilitate transmission at the individual or population level [33–35].

Several studies have utilized full-genome sequences to evaluate norovirus transmission and evolution in different settings. Thus, full-genome sequences of noroviruses have proven to be instrumental in assessing the diversification and the direction of transmission during nosocomial infections [36–40] and given geographical regions [41–43]. Importantly, some of these studies have shown that, in addition to changes in the major structural protein, VP1, and the viral RNA polymerase, other viral proteins (like NS1/2 or VP2) might play an important role in the transmission and emergence of novel noroviruses [30,42].

Despite almost two decades of robust sequence and epidemiological norovirus studies, such full-length genome analyses are scarce and genomic regions other than VP1 and NS7 have been largely understudied. Moreover, most norovirus genome records available were obtained from predominant genotypes that circulated since the mid-2000s in developed countries. Thus, there is a large data gap on the spatiotemporal genomic diversification of human noroviruses. To gain insights on the evolution and mechanisms of norovirus emergence, we collected archival samples and sequenced over 281 viruses circulating since the 1970s in over 10 countries from four continents, with a major emphasis on those genotypes that are underrepresented in the public databases. Thus, using this improved dataset of human norovirus genomes we aimed to (i) revisit evolutionary parameters at the genome-wide level that could provide insights on norovirus diversification, (ii) perform a detailed analysis of the phylogenetic relationship among atypical and untypeable viruses, and (iii) investigate factors that govern recombination among noroviruses.

## Results

**Fig 1:**
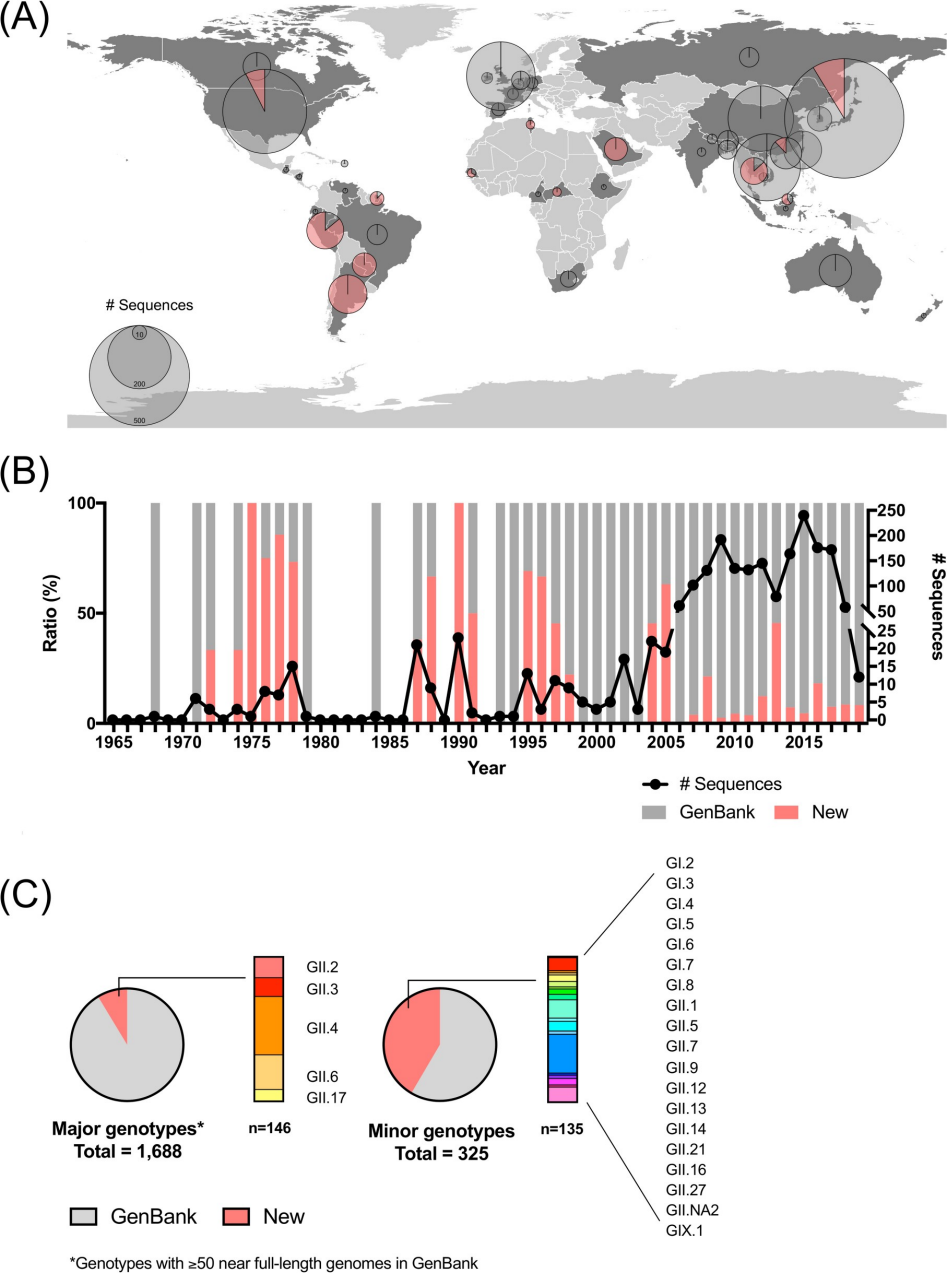
Spatiotemporal distribution of norovirus genomes analyzed in this study. (A) Geographic map showing the countries with nearly full-length genome sequences highlighted in dark gray. The pie chart indicates the ratio of newly obtained sequences in this study (red) and those from the public database, GenBank (gray). The size of the pie chart shows relative sample size for each country. Geographic map (1:10m Cultural Vector, Admin 0 –Countries) was obtained at https://www.naturalearthdata.com/downloads/10m-cultural-vectors/10m-admin-0-countries/ (accessed on June 25, 2021). (B) The line indicates the number of nearly full-length genome sequences by year and the bar graph indicates the ratio of newly obtained sequences (red) and public database (gray). (C) Number of newly obtained sequences for major and minor genotypes. The pie chart indicates the ratio of newly obtained sequences in this study (red) and those from public database, GenBank (gray).

We retrieved 1,732 nearly full-length human norovirus genomes available in GenBank as of March 30, 2020. While genomic data on norovirus have drastically increased during the last decade (1,175 out of 1,732 deposited sequences), minimal information (62/1,732) was available for noroviruses collected before 2000. Moreover, most genome sequences (1,542/1,732) corresponded to viruses from six genotypes (GII.2, GII.3, GII.4, GII.6, and GII.17). In this study, we sequenced 281 new nearly full-length genomes of human noroviruses collected globally from 1972 to 2019 (Fig 1 and S1 Table). The viruses sequenced here were mostly sampled from geographic regions and decades with scarce information. Our current sequencing efforts include an expansion of the geographical information, with a particular increase in the number of norovirus genomes for South America (27 to 169 genomes), Africa (14 to 22 genomes), and Asia (1,082 to 1,169 genomes), the first two geographic regions with very under-represented norovirus genomic information (Fig 1A). Our efforts also increased the number of genomes for noroviruses sampled before 2000 (62 to 144 genomes) (Fig 1B), providing the oldest genome information from 12 norovirus genotypes (Table 1), and new genomes for genotypes with under-represented genomic information (Fig 1C). Thus, this study provides more than 42% (135/325) of the genome information available for those under-represented human norovirus genotypes (Fig 1C), with new nearly full-length genomes for genotypes GII.9, GII.27 [44], and GII.NA2 [45]. Together with the genomes retrieved from GenBank, our analyses included a dataset of 2,013 human norovirus genomes.

**Table 1:** Oldest nearly full-length sequences from each genotype.

Genotype*	Polymerase type*	Virus	Year	Accession number
GI.1	GI.P1	8FIIa	1968	M87661
GI.2	GI.P2	Southampton	1991	L07418
GI.3	GI.PCNA	B8	1976	MW305491 (in this study)
GI.4	GI.P4	Chiba407	1987	AB042818
GI.5	GI.P12	SzUG1	1999	AB039774
GI.6	GI.P6	BS5-HSS-3	1997	AF093797
GI.7	GI.P7	DS386	1990	MW305508 (in this study)
GI.8	GI.P8	Nagoya-KY531	2007	KJ196298
GI.9	GI.P9	CAIQ12110628	2012	KF586507
GII.1	GII.P1	Hawaii	1971	U07611
GII.2	GII.P32	HenrytonSP17	1971	MF405169
GII.3	GII.P41	ShippensburgB24^**#**^	1972	KY442319
GII.4	GII.P39 and GII.PNA3	CHDC5191/CHDC2094^**#**^	1974	JX023286/FJ537135
GII.5	GII.P22	C15^**#**^	1978	MW305580 (in this study)
GII.6	GII.P6	HenrytonH1^**#**^	1971	KY424345
GII.7	GII.P36	HK4	1976	MW305573 (in this study)
GII.8	GII.P8	Saitama_U25	1998	AB039780
GII.9	GII.P7	PNV008798^**#**^	2008	MW305514 (in this study)
GII.10	GII.P12	Vietnam_026	1999	AF504671
GII.12	GII.P29	110–2^**#**^	1987	MW305595 (in this study)
GII.13	GII.P41	KL90	1978	MW305577 (in this study)
GII.14	GII.P7	B17-c1	1976	MW305569 (in this study)
GII.16	GII.P16	Neustrelitz260	2000	AY772730
GII.17	GII.P13	T055	1977	MW305582 (in this study)
GII.20	GII.P20	Leverkusen267	2009	EU424333
GII.21	GII.P21	YO284	2007	KJ196284
GII.22	GII.P22	YURI	2003	AB083780
GII.23	GII.P23	PE1848	2010	KY496328
GII.24	GII.P24	Loreto7940^**#**^	2013	MN453359
GII.25	GII.P25	Dhaka1928	2012	MG495083
GII.26	GII.P26	Leon4509	2005	KU306738
GII.27	GII.PCNA	PNV024019^**#**^	2010	MK733206 (in this study)
GII.NA1	GII.PNA1	Loreto1257	2013	MG495079
GII.NA2	GII.PNA2	PNV06929^**#**^	2008	MG706448 (in this study)
GIX.1	GII.P15	DS335^**#**^	1990	MW261800 (in this study)
GVIII.1	GII.P28	Yuzawa-Gira2HS	2011	KJ196291

*CNA: Could not be assigned, NA: Not assigned

^#^Only one representative virus is listed here.

### Near full-length archival samples improve the estimates of evolutionary parameters

**Fig 2:**
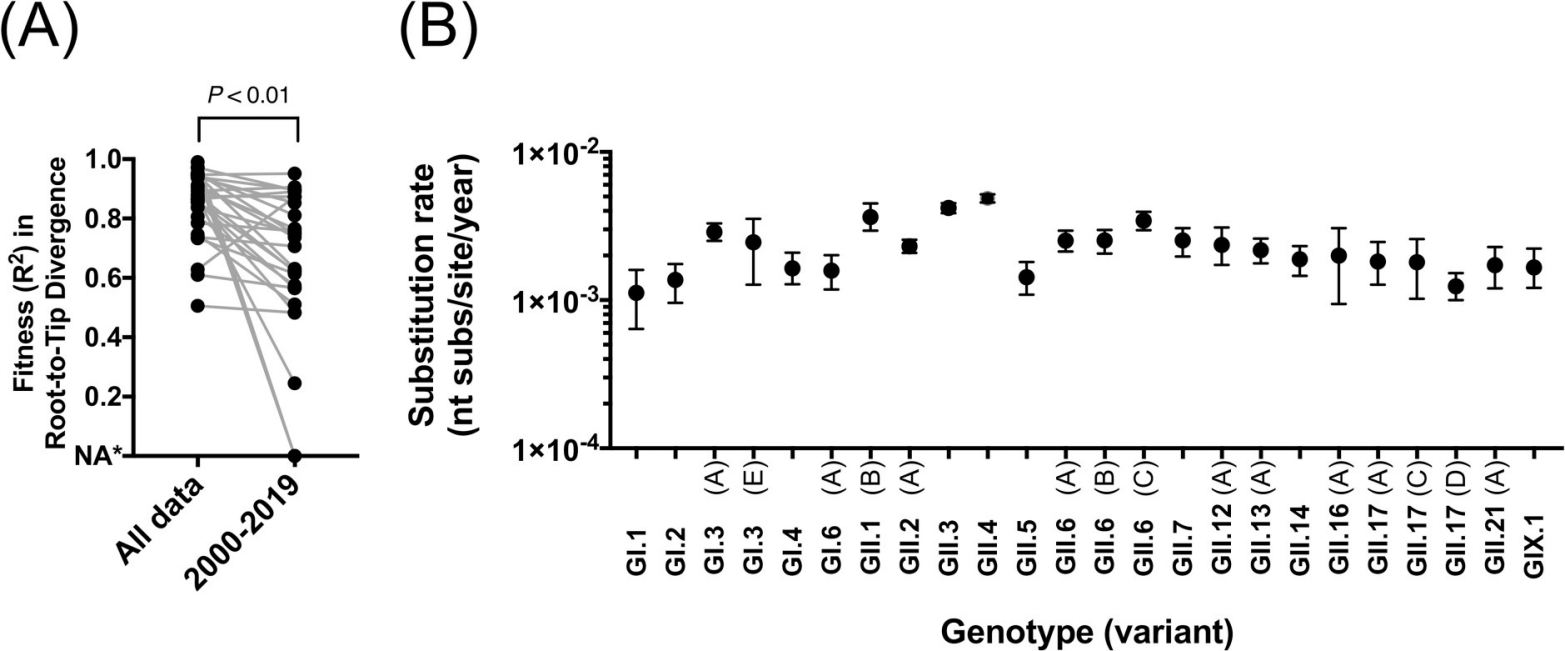
Archival samples improved the estimates of the rate of evolution. (A) Linear regression of the root-to-tip divergence of the VP1-encoding nucleotide sequences indicated the improvement of the fitness (R^2^) by adding the archival samples in the analyses (P<0.01 in Wilcoxon matched-pairs signed rank test). The R^2^ values were calculated using maximum-likelihood phylogenetic trees from each genotype or variant. *NA indicates not enough sequences to build phylogenetic trees. Genotypes or variants presenting >5 sequences and time range >5 years were included in the analyses. (B) Mean and the 95% highest posterior density interval of nucleotide substitution rate (substitutions/site/year) were estimated for each genotype and variant with Bayesian framework. Only genotypes or variants presenting >10 sequences and time range >5 years were included in the analyses.

Due to the evolutionary patterns presented by the different norovirus genotypes [14,46], absence of viruses circulating in different decades could result in inconsistencies on the estimates of evolutionary rates [21], particularly for those genotypes presenting different variants [46]. Thus, because our work provided the oldest genome information for 12 out of the 36 genotypes described for human norovirus, we thought to calculate the evolutionary rates using this novel information. We found that inclusion of historical samples collected before the 1990s significantly improved the clock-likeness of the VP1-encoding sequences in the dataset (P<0.01 in Wilcoxon matched-pairs signed rank test, Fig 2A). The R^2^ from VP1 phylogenetic root-to-tip regression was improved in correlation with longer duration of years analyzed, providing the accuracy of evolutionary rates estimated. Overall, the rate of evolution was similar for most of the human noroviruses (1.12–4.86 × 10^−3^ nucleotide substitutions/site/year), with the 95% highest posterior density (95%HPD) intervals overlapping among most of the genotypes/variants (Fig 2B and S2 Table). Some of the 95%HPD intervals did not overlap with others, suggesting small differences among the rate of evolution of distinct genotypes. The highest evolutionary rate on VP1-encoding sequences was recorded in GII.4 viruses, which presented periodic emergence and replacement of variants [12]. Some non-GII.4 viruses, e.g. GI.3, GII.6, and GII.17, could be also divided into variants using a cutoff of 5% amino acid differences in the VP1. Notably, while GII.4 variants circulated only for a short period (3–8 years), most non-GII.4 variants have been shown to co-circulate for decades [14,46]. The distinct non-GII.4 variants presented only minor differences in the rate of evolution, but that difference did not correlate with the prevalence and epidemic potential of viruses.

**Fig 3:**
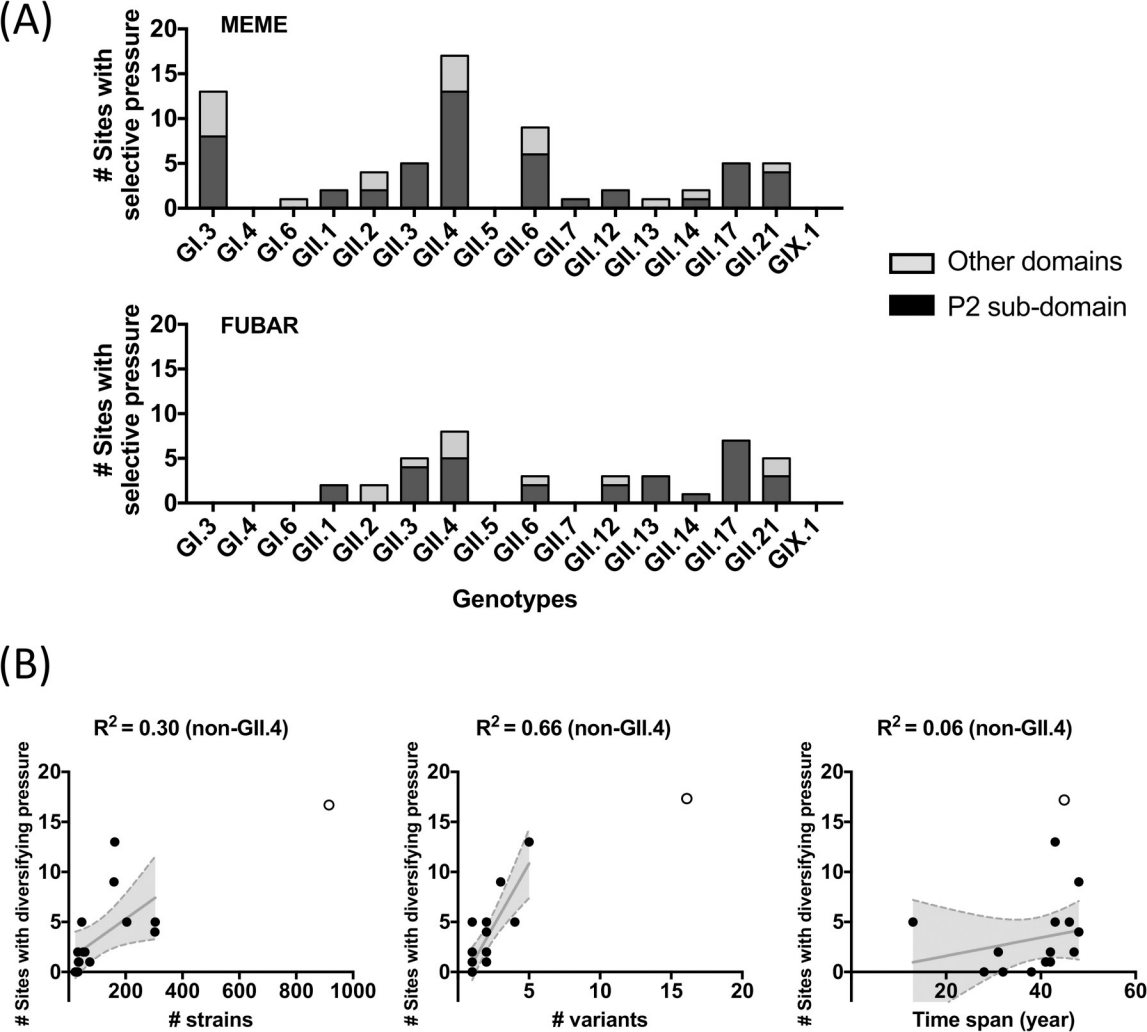
Diversifying selection on the major capsid protein (VP1) results in multiple intra-genotype variants. Episodic diversifying selections were comprehensively estimated for GI, GII, and GIX genotypes presenting ≥20 sequences using MEME (Mixed Effects Model of Evolution), which modeled branch-by-branch episodic pressure, and FUBAR (Fast, Unconstrained Bayesian AppRoximation), which assumed constant pressure across the entire phylogenetic tree. (A) Statistically significant positively selected sites (P<0.05 with empirical Bayes Factor on internal branches>100 in MEME and Bayes Factor >0.9 in FUBAR) were counted in each genotype and summarized as bar plot. (B) The excessive number of sites identified as being under episodic diversifying selection on non-GII.4 viruses was correlated with the number of variants but not with the number of sequences or time span. The linear regression line and the 95% confidence band were plotted in gray. Circles represent non-GII.4 (filled) and GII.4 (empty) genotypes.

**Fig 4:**
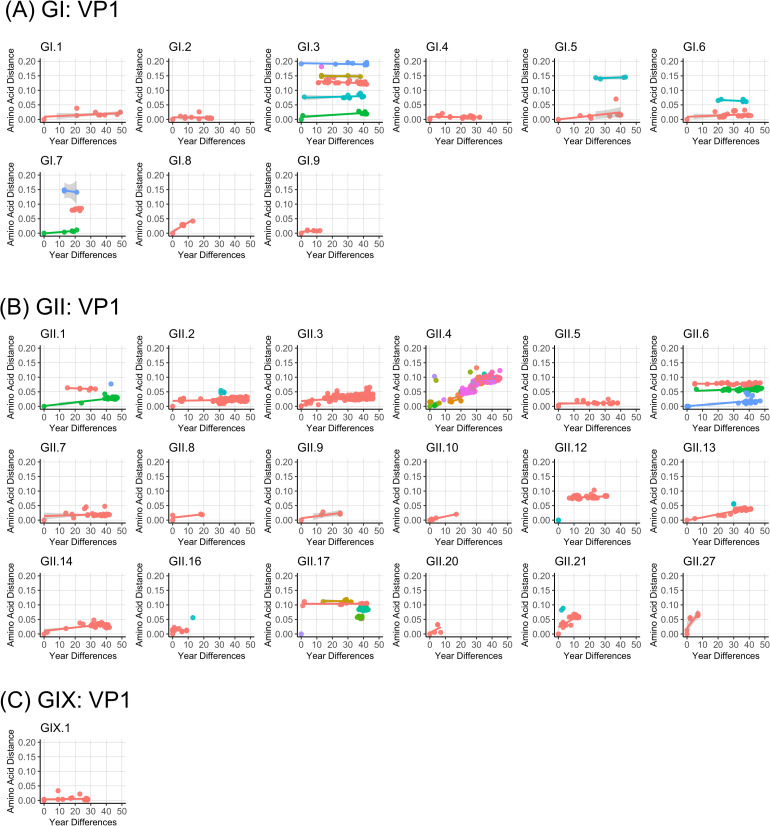
Limited accumulation of amino acid mutations on the major capsid protein of non-GII.4 noroviruses. Amino acid distance was calculated from the oldest viruses for each given genotype from (A) GI, (B) GII, and (C) GIX viruses. Only genotypes with data from samples with ≥5 sequences were analyzed. Variants within each genotype were separately analyzed and are shown with different colors. Lines represent the linear regression for amino acid mutations occurring during a given time span for each genotype or variant.

Diversifying selection on the VP1 protein was recorded in major genotypes, with GII.4 viruses presenting the largest number of sites under positive pressure (Fig 3A). Most of these residues were mapped on the P2 sub-domain, as previously shown [16]. Non-GII.4 genotypes, i.e. GI.3, GII.3, GII.6, GII.17, and GII.21, also presented a large number of sites identified as being under positive pressure, which seems correlated with the number of variants (R^2^ = 0.66 in a linear regression) rather than the number of sequences (R^2^ = 0.30) or time span available (R^2^ = 0.06) (Fig 3B). Accordingly, the estimated number of sites under positive pressure decreased sharply when looking into individual variants, with a smaller number of sites on P2 domains identified to be under pressure (S1 Fig). Notably, despite the large number of sites were identified as under positive pressure on those non-GII.4 genotypes, none of them accumulated amino acid differences on VP1 as compared to GII.4 noroviruses (Fig 4) [14].

**Fig 5:**
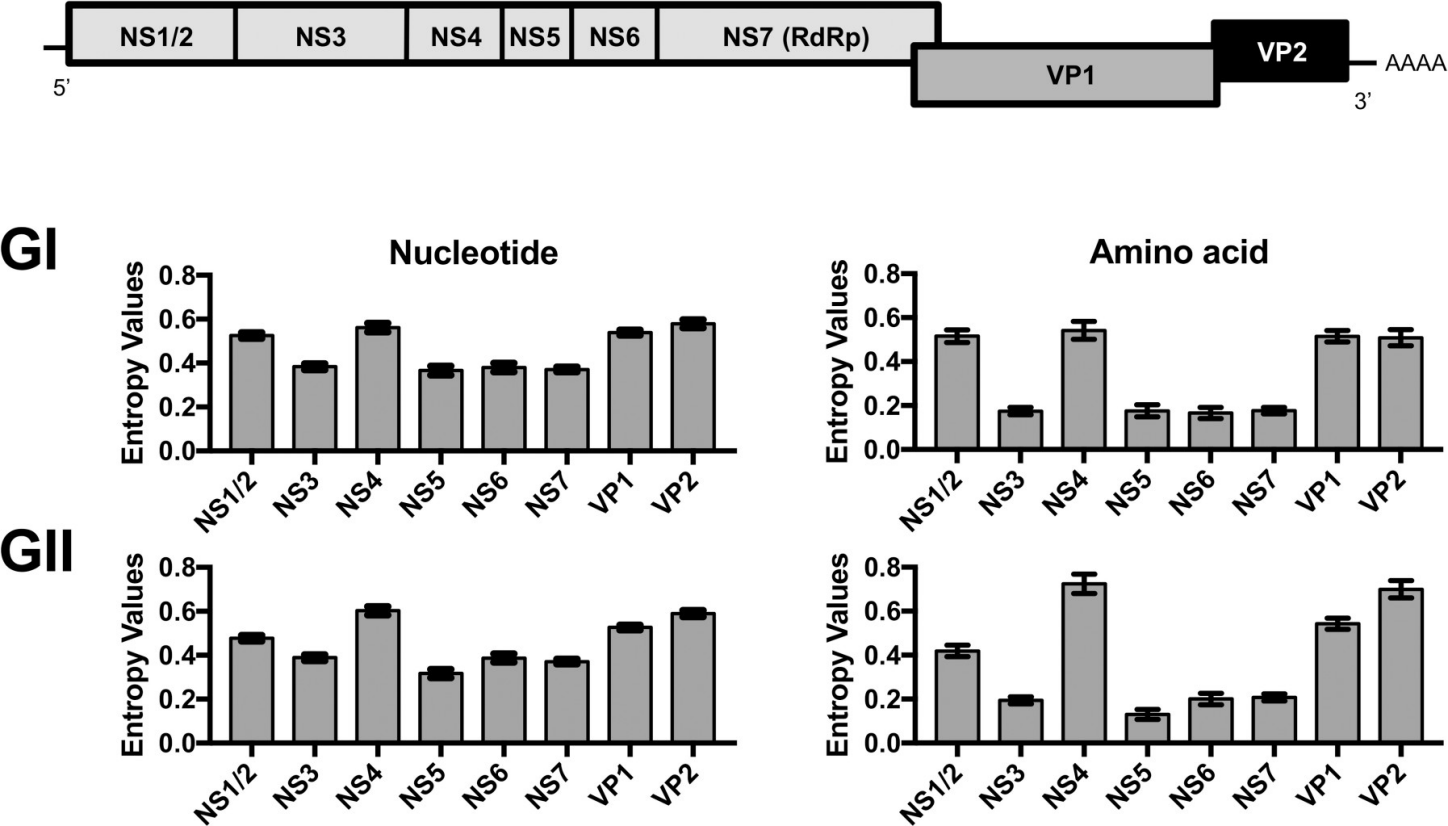
Genome-wide analysis indicates that NS1/2, NS4, major (VP1), and minor (VP2) capsid proteins are the most variable regions of human noroviruses. Genetic variability of encoding regions of nonstructural (NS1/2–7) and capsid (VP1 and VP2) proteins was calculated using Shannon entropy for GI and GII noroviruses. Bars represent the mean value calculated from individual residues values. Standard errors are shown for each bar.

**Fig 6:**
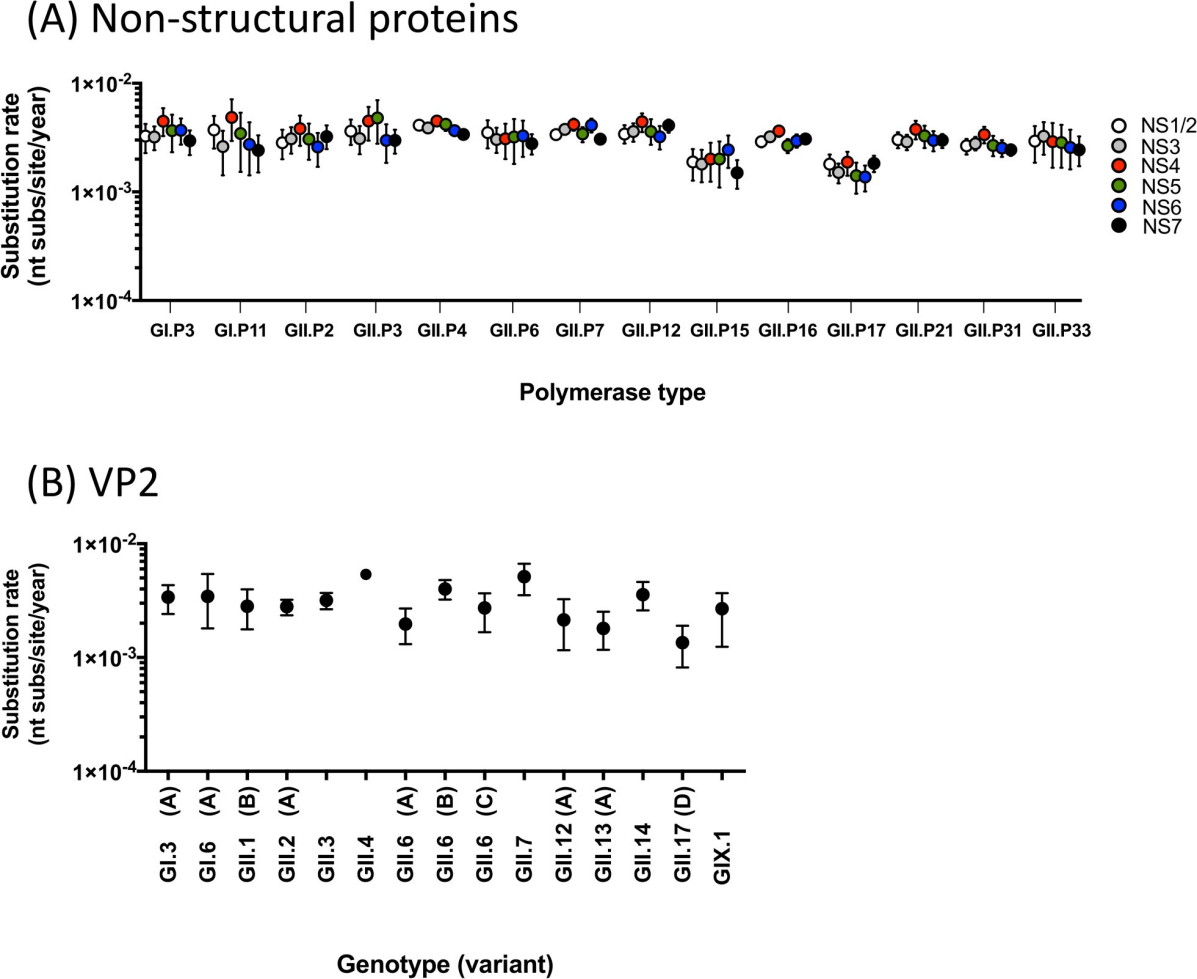
Noroviruses evolve at similar rates across genomic regions and genotypes. The mean and the 95% highest posterior density interval of nucleotide substitution rate (substitutions/site/year) were estimated from sequences encoding (A) non-structural and (B) VP2 proteins. Only genotypes or variants presenting >10 sequences were included in the analyses.

Genome-wide analyses of GI and GII viruses revealed that in addition to VP1, NS1/2, NS4, and VP2 proteins presented significantly higher diversity at both nucleotide and amino acid level (P<0.05 in one-way ANOVA with post hoc Tukey multiple-comparison test, Fig 5). The amino acid differences have been accumulating over time in non-structural proteins, such as NS4 and NS7, and VP2 proteins with the high/low regression slope reflecting its overall diversity (S2–S4 Figs). Notably, there were no significant differences in the diversification pattern of those proteins between GII.4 and non-GII.4 viruses. The substitution rate of nucleotide sequences from non-structural proteins and VP2-encoding sequences showed an overall similar rate among genome regions and polymerase types/genotypes (Fig 6), which was within the same range as those from VP1 sequences.

### Differences in non-structural proteins between endemic and epidemic viruses

As the VP1 protein is the major target of immune responses, changes on this protein could be a key driver of viral transmission [16,47]. However, while some noroviruses emerge and transiently predominate globally (epidemic), other viruses with the same polymerase and/or capsid types only circulated at low levels (endemic) or were associated with geographically- and temporally-restricted outbreaks. Two of such examples are: (i) the GII.2[P16] viruses that predominated in some Asian and European countries in the 2016/2017 winter season without major changes on its ORF2 (capsid) sequences, and (ii) the GII.4 Sydney 2012 viruses with GII.P31 polymerase type that emerged and predominated globally since 2012 [48]. Thus, we decided to investigate the differences in the entire ORF1 from (i) epidemic GII.P16 viruses compared with those endemic GII.P16 viruses, and (ii) epidemic GII.P31 (GII.4 Sydney 2012) viruses compared with those endemic viruses (e.g. GII.4 Osaka 2007 variant, GII.17 variant A, and GII.2) presenting a similar GII.P31 polymerase type (S5A Fig). The phylogenetic trees along with ancestral sequence reconstruction indicated multiple amino acid mutations on the NS4 and NS7 that differentiated the endemic and epidemic viruses on the trees. Other non-structural proteins did not possess specific residues that could separate epidemic and endemic viruses on the trees and those viruses were distributed on the same branches. An exception to this was present at the NS1/2 protein from GII.P16 viruses, which presented a mutation (N52E/K) and insertions of one or two glutamic acid (E) at residue 77 [49] in the epidemic viruses. Notably, 6 out of 7 amino acid mutations on the RNA polymerase that differentiate epidemic viruses from endemic viruses (residues 173, 293, 357, 360 for GII.P16 [23,30]; 4 and 236 for GII.P31) mapped on the surface of the protein (S5B Fig).

The dominance of GII.4 viruses has been explained by chronological emergence of six variants: Grimsby 1995, Farmington Hills 2002, Hunter 2004, Den Haag 2006b, New Orleans 2009, and Sydney 2012, with the first five presenting a GII.P4 polymerase type. In addition, GII.4 also presents so-called minor variants that express the GII.P4 polymerase, namely: Sakai 2003, Yerseke 2006a, Osaka 2007, and Apeldoorn 2007. These minor variants are characterized by their limited epidemiological impact and spatiotemporal distribution. We systematically searched for mutations on the phylogenetic trees from GII.P4 non-structural proteins that differentiate these variants from those considered epidemic variants; however, neither phylogenetic signals nor amino acid mutations at the non-structural proteins were able to differentiate the epidemic from endemic GII.P4 viruses (S6 Fig).

Interestingly, the phylogenetic diversification of VP2 proteins revealed differences among epidemic and endemic GII.2 and GII.4 viruses (S7 Fig). The epidemic GII.2[P16] viruses experienced one amino acid mutation (G/S109N) on the VP2 as compared to those endemic GII.2[P16] viruses (S7A Fig). Meanwhile, the phylogeny of the VP2 protein from GII.4 viruses resembled that of VP1, in which a distinct clustering was observed for each of the VP1 variants (S7B Fig); however, the endemic viruses in most cases grouped with the major epidemic variants. Examples of those include viruses from endemic GII.4 Osaka 2007 variant that were distributed in the branches from the epidemic Den Haag 2006b variant—possibly due to a recombination at ORF2/ORF3 region [50], and those from Apeldoorn 2007 clustered together with the New Orleans 2009 variant. Moreover, the recently described Hong Kong 2019 variant [51] presented a VP2 protein that is relatively close to the Grimsby 1995 variant and a GII.P31 polymerase that clustered with non-epidemic viruses. The epidemiological relevance of the Hong Kong 2019 variant, and associated genes, is currently unclear but will be revealed in the future. Together, we found multiple mutations on NS1/2, NS4, and NS7 proteins that could differentiate the epidemic and endemic viruses. However, none of those mutations were detected in non-structural proteins from epidemic GII.4[P4] viruses, indicating that these different residues could have emerged due to founder effect and/or not by contributing to epidemic potential [12].

### Analyses of intermediate, minor, and unique viruses

**Fig 7:**
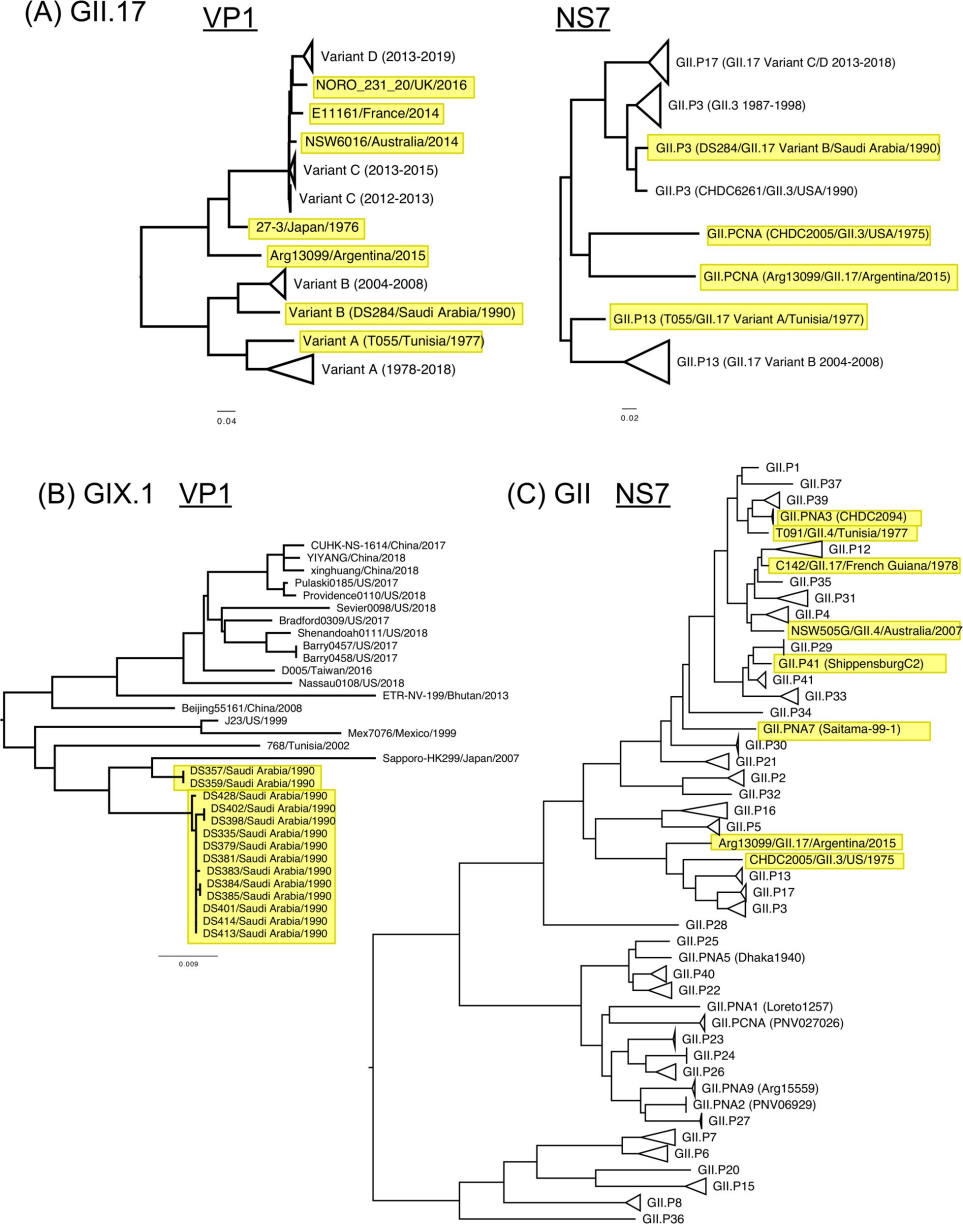
Large-scale phylogenetic analyses revealed intermediate or minor viruses. Large-scale phylogenetic trees from (A) GII.17 VP1 and NS7, (B) GIX.1 VP1, and (C) GII NS7 nucleotide sequences revealed atypical viruses embedded in their evolutionary history. Newly described viruses and intermediate viruses are highlighted in yellow. Genotypes and variants in clusters were summarized and collapsed for visualization.

In addition to contributing archival full-length genome information of major and minor human norovirus genotypes, our study also focused on expanding the information for atypical viruses, as those could provide valuable information to our understanding of the epidemiology and/or evolution of human noroviruses. Thus, we sequenced a GII.17 virus (Arg13099) detected in 2015 in Argentina that did not cluster with any of the variants described for the VP1 [52] (Fig 7A). We also found in our dataset two archival GII.17 viruses (T055/Tunisia and DS284/Saudi Arabia from 1977 and 1990, respectively) that were indeed intermediate, and did not cluster with the rest of the viruses of each given variant on the VP1 tree. To gain insights on the diversification of GII.17 viruses, we also analyzed the NS7 sequences from those viruses. The Arg13099 virus did not cluster with any polymerase type, and the T055 virus was typed as GII.P13, but situated outside of the cluster that included other GII.P13 viruses (Fig 7A). DS284 was the only GII.17 virus that presented a GII.P3 polymerase. Because GII.P3, GII.P13, and GII.P17 NS7 sequences share a common ancestor and are all genetically close, it is difficult to determine whether the DS284 virus is a recombinant or another lineage. Other intermediate viruses from GII.17 were also reported in the public database, e.g. 27-3/Japan, NORO_231_20/United Kingdom. Thus, these different intermediate viruses suggest that the GII.17 virus explored multiple variants (and phenotypes) until reaching epidemic potential in 2014.

Because of the very low incidence in the human population, GIX.1 (formerly GII.15) viruses are regarded as a minor genotype. This genotype has not been reported to cause large outbreaks and is only detected sporadically in limited studies [53–55]. However, when analyzing samples from US troops deployed to Saudi Arabia in 1990, Desert Shield operations, that presented gastrointestinal symptoms [56], we detected 14 GIX.1 viruses in 22 of the samples successfully sequenced from this outbreak (Fig 7B). Two distinct transmission chains of GIX.1 viruses were observed in this outbreak, which presented 37–39 nucleotide differences on the VP1 among the viruses from those two transmissions; only two of them were nonsynonymous mutations. Overall, the GIX.1 genotype presented very limited diversity on its VP1 and the corresponding GII.P15 polymerase for the past 30 years (Figs 4 and S3). The rest of the samples from the Desert Shield outbreak were characterized as the prototype GI.3 virus (DSV395; Desert Shield virus), GI.7, GII.3, GII.14, and GII.17. This finding suggests that viruses belonging to the GIX.1 genotype could also be linked to large gastrointestinal outbreaks [57].

During our study, we also found novel viruses (recently assigned as GII.26, GII.27, and GII.NA2) [44,45]. Notably, these viruses belonging to different genotypes clustered together and most of them were detected in different countries from South America [44]. One of the viruses was a recombinant with a VP1 sequence from GII.12, and all of them presented similar VP2 sequences with those from GII.3 viruses [44] and from one GII.2 virus (GII.2/OsakaNI) (S8 Fig; details described in the next section).

Finally, multiple untypeable viruses were detected when their NS7 were analyzed (Fig 7C). While the NS7 protein showed less variation as compared with VP1 sequences (Fig 5), the untypeable viruses were placed on the tree as intermediates of the evolution among polymerase types. In norovirus nomenclature, the different genotypes are assigned by a tight phylogenetic clustering of viruses, and recombinant viruses are mostly recognized by presenting a VP1 capsid genotype and a different polymerase type. Thus, these intermediate viruses make the polymerase clustering less distinct, and the typing of novel strains more challenging.

### Restrictions on ORF1/ORF2 norovirus inter-genotype recombination

**Fig 8:**
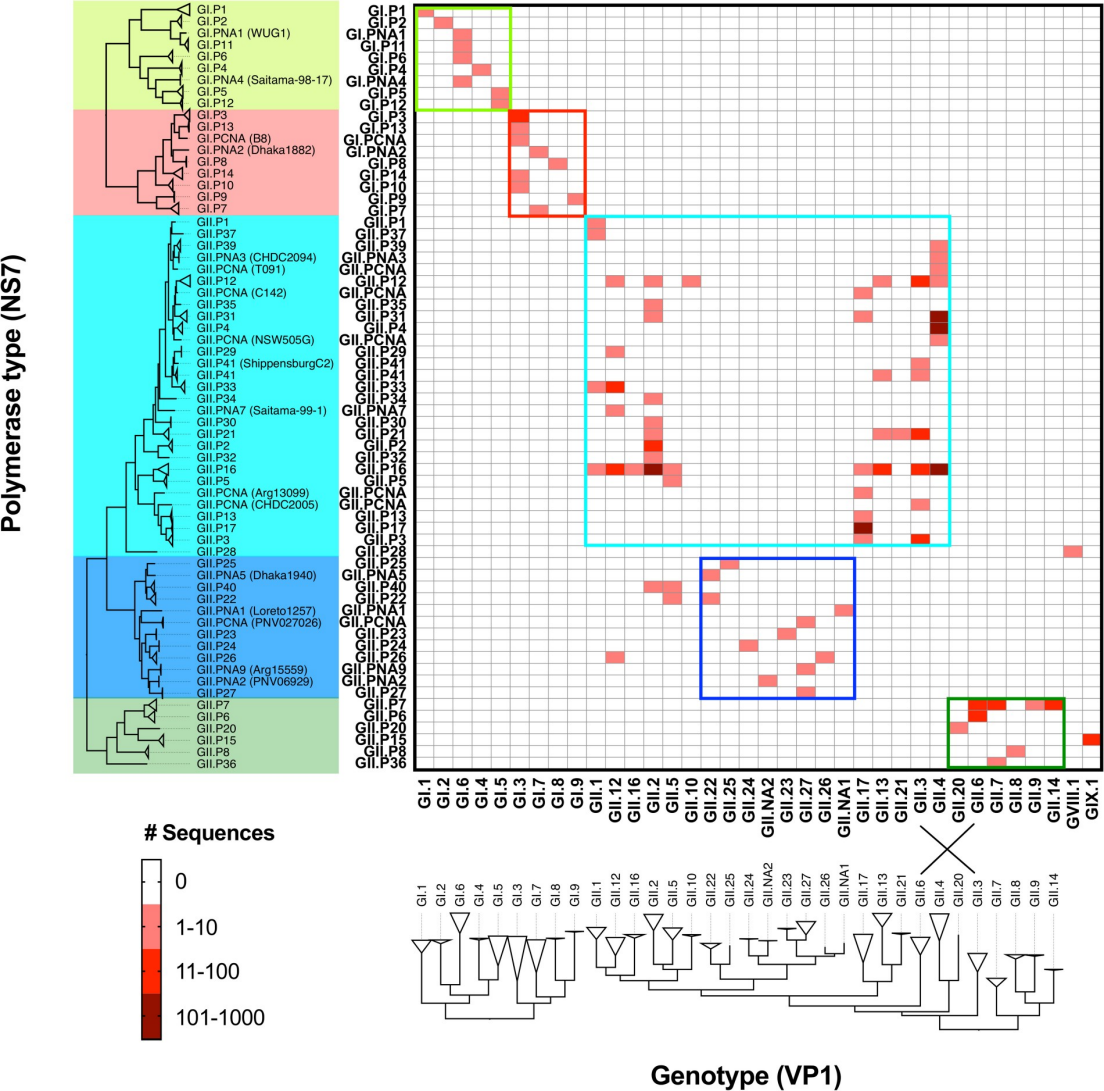
Phylogenetic pairing of capsid and polymerase types suggests restriction on recombination. Genotypes were determined for all the viruses in the nearly full-length dataset using Norovirus Genotyping Tool [102]. Viruses were grouped phylogenetically and the number of viruses with a given capsid and polymerase types was recorded in each cell. Phylogenetic trees were calculated using a subsampled dataset: a maximum of two viruses from each combination of genotype and polymerase type. The colored boxes in the matrix indicate the recombination groups associated with the phylogenetic clustering on the NS7-encoding nucleotide sequences. The crossing lines on the phylogenetic tree of the VP1 indicate the disruption of VP1- clustering and recombination groups.

Recombination is one of the most important aspects of the molecular epidemiology of noroviruses [27]. A large number of recombinant viruses has been reported in the literature [28,29], but only a few are regarded as epidemiologically relevant. Some of those are GII.4 Sydney 2012[P31], GII.4 Sydney 2012[P16], GII.2[P16], GII.6[P7], and GII.3[P12] [17,32,48,50,58,59]. Based on the number of genotypes and polymerase types, recombination at the ORF1 and ORF2 junction region could theoretically generate >1200 capsid-polymerase combinations; however, in our nearly full-length database we only found 92 capsid-polymerase combinations, including 18 new combinations that were confirmed with full-length genome sequences in this study (Table 2). To gain insights on the mechanisms behind norovirus recombination, we first organized and tabulated all capsid and polymerase types included in our dataset based on their phylogenetic clustering (Fig 8). Notably, the phylogeny from NS7 presented five major clusters, each including several polymerase types (Fig 8), that were associated with a specific subset of capsid genotypes. Thus, the GI genotypes presented two distinct clusters at the NS7 and VP1 tree, and recombination was detected only among genotypes from the same clusters. Similarly, the GII polymerase types showed one large and two small clusters, and recombination was restricted to capsid genotypes linked to the viruses grouped in the same NS7 cluster. One of the smaller groups included viruses presenting the GII.P6, GII.P7, GII.P8, GII.P20, and GII.P36 polymerase that were linked only to the GII.6, GII.7, GII.8, GII.9, GII.14, or GII.20 capsid genotypes, while the other small group included recently described genotypes (GII.22-GII.27) and polymerase types (GII.P22-GII.P27, GII.P40). The latter could be embedded within the larger group that include all other GII viruses because of the phylogenetic clustering and a limited number of viruses from three genotypes (GII.2, GII.5, and GII.12) presenting the newly described polymerase types (Fig 8). Notably, GII.P15 and GII.P28 did not follow the recombination restriction, as they were associated with only the capsid from non-GII viruses, GIX.1 and GVIII.1, respectively. Viruses presenting these combinations confirm the possibility of inter-genogroup recombination; however, no parental viruses have been detected so far.

**Table 2:** Newly obtained capsid-polymerase combinations with nearly full-length sequences.

Genogroup	Genotype [polymerase type]*	# Sequences	Accession numbers^#^
GI	GI.3[P10]	2	MW305506
	GI.3[PCNA]	1	MW305491
	GI.5[P5]	2	MW305487
GII	GII.2[P21]	2	MW305600
	GII.2[P34]	1	MW305657
	GII.2[P35]	1	MW305570
	GII.3[PCNA]	1	MW305724
	GII.5[P22]	2	MW305580
	GII.7[P36]	1	MW305573
	GII.9[P7]	3	MW305514
	GII.12[P26]	1	MK733201
	GII.12[P29]	4	MW305593
	GII.13[P41]	1	MW305577
	GII.17[P3]	1	MW305734
	GII.17[P13]	8	MW305512
	GII.27[PNA9]	4	MK733202
	GII.27[PCNA]	2	MK733206
	GII.NA2[PNA2]	2	MG706448

*CNA: Could not be assigned, NA: Not assigned.

^#^Only one representative virus is listed here.

GI and GII phylogenetic trees from each of the non-structural protein-encoding sequences showed similar topologies (S8 Fig). In both genogroups, the NS7 clusters (color-shaded in S8 Fig) were preserved in other non-structural proteins, suggesting a limitation for recombination within the ORF1. The blue cluster in GII was nested in the light blue cluster in the NS6 phylogenetic tree, but still presented distinct separation from each other. Those clusters were disrupted in the VP1- and VP2-based trees in GII (S8 Fig). Together, NS7 (or ORF1-based) clusters were associated with the restriction and pattern of recombination (recombination group).

**Fig 9:**
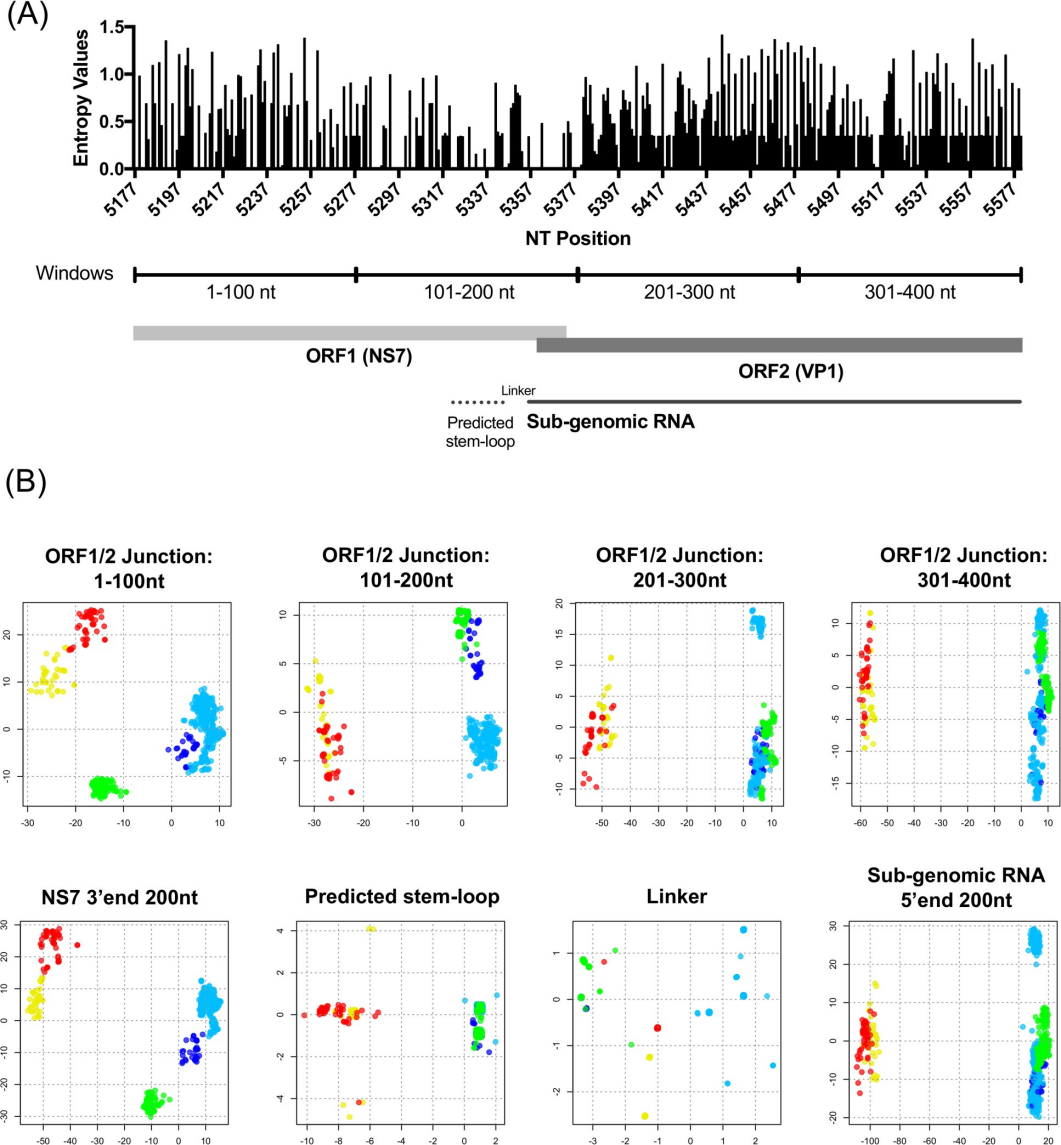
Recombination is restricted by sequence variation at the 3’ end of the ORF1 region. (A) Genetic variability of the ORF1/2 junction region (400 nt) was measured using Shannon entropy. Stem-loop sequences on predicted sub-genomic RNA promoter and sub-genomic RNA was determined based on analyses from Simmonds et al. [60]. Nucleotide position was recorded based on Norwalk virus 8flla (accession number M87661). (B) Multidimensional Scaling Analysis (MDS) revealed the association between recombination pattern and nucleotide sequence variation at the 3’ end of ORF1 region. MDS (two-dimensional) was conducted with four non-overlapping windows in the ORF1/2 junction (400 nt): 1–100 nt and 101–200 nt in the 3’ end of ORF1, and 201–300 nt and 301–400 nt in the 5’ end of ORF2 region (top panels). MDS was also provided with different regions based on their genetic functions: 3’ end of NS7, predicted stem-loop, linker sequence, and 5’ end of sub-genomic RNA (bottom panels). Each dot represents a virus sequence color-coded based on the respective recombination group as defined in Fig 8. The MDS was conducted using nucleotide differences among viruses and the two dimensions in the MDS maps were represented by x- and y- axes with their directions arbitrary determined.

To determine the mechanisms associated with the restrictions on the observed ORF1/ORF2 recombination, we examined the diversification pattern of different regions adjacent to the ORF1/ORF2 junction region. Thus, we examined 200 nt from the 3’-end of the NS7 (ORF1) and 200 nt from the 5’-end of the VP1 (ORF2). This junction region is highly conserved [27], particularly within the ~20 nt encompassing the ORF1/2 overlapping region (Fig 9A). To explore the sites correlated with groups presenting recombination restriction, we performed a multidimensional scaling (MDS) analysis using nucleotide sequence variation at the ORF1/2 junction (Fig 9B). The 400 nt junction region was divided into four windows, each corresponding to 100 nt in length. The first two regions correspond to the 3’-end of ORF1, and the last two correspond to 5’-end of ORF2 sequences (Fig 9A). These MDS maps indicated strong clustering in the first window (Fig 9B), while the second window presented modest clustering by recombination group. On the other hand, the two windows from the 5’-end of ORF2 showed strong clustering by genogroups, but not by recombination groups. The MDS maps were also created from genome regions by their function: namely 3’end of ORF1 (NS7), 5’end of ORF2 (sub-genomic RNA), predicted stem-loop, and linker sequence between predicted promoter core and sub-genomic RNA [60] (Fig 9B, bottom panels). Recombination groups were only reproduced from MDS analysis done with the 3’ end of the ORF1, suggesting that recombination occurs only between viruses that shared similar 3’ end on their ORF1 sequences. Both stem-loop and 5’ end of sub-genomic RNA sequences clustered by genogroups, and there was no specific clustering in the linker sequences. We further looked into specific polymerase types and capsid genotypes with multiple recombinants reported, i.e. GII.P7, GII.P16, GII.2, GII.3, and GII.4 viruses, which are the most prevalent viruses in the different recombination groups (S9 Fig). Those viruses were marked in black on the same MDS maps in Fig 9. While ORF2-based MDS maps (201–300 and 301–400 nt windows) did not show distinct clusters in GII.P7 and GII.P16 viruses, ORF1-based MDS maps presented distinct clusters in all of the five viruses regardless of the counterpart polymerase types and capsid genotypes of recombination, supporting the important role of sequence similarity at the 3’ end of ORF1 on recombination.

### Limited evidence of intra-genotype recombination in intra-host viral populations

Intra-genotype recombination requires mixed-infections in the host followed by co-infection of cells [28,61]. One advantage of the full-length sequencing platform presented here is that we utilized full-length PCR amplicons and next-generation sequencing (NGS) technology [14], which enabled us to detect reads from different viruses in a given sample.

**Fig 10:**
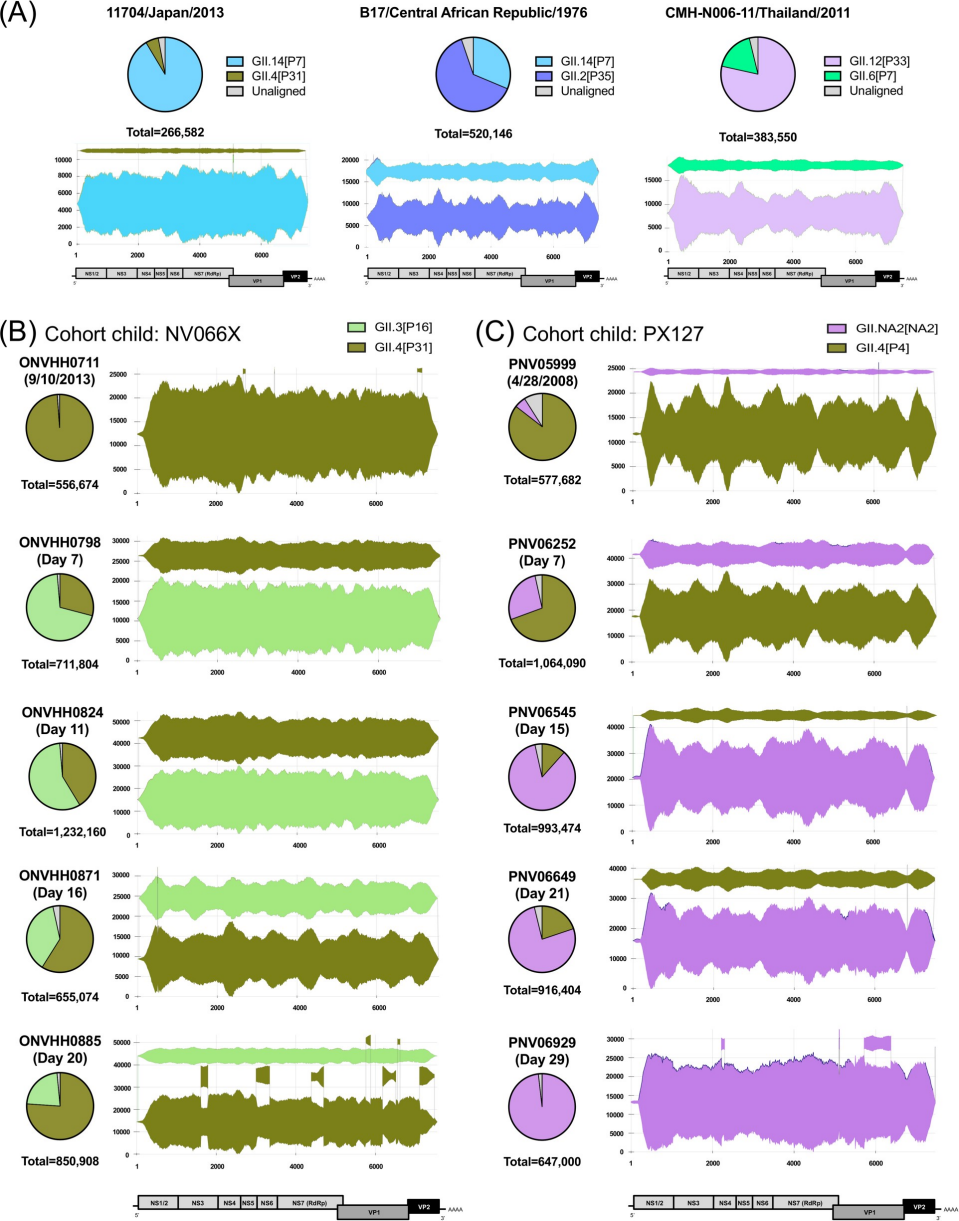
Mixed-infections with multiple norovirus genotypes suggest limited recombination events. (A) Three cross-sectional cases and (B and C) two prolonged shedding cases for cohort children (NV066X and PX127, respectively) of mixed infection with two different norovirus genotypes. Pie charts represent the ratio of the read counts from each genotype, with total number of reads shown on the bottom of the pie charts. The Sanky diagrams represent the assembled contigs (clones) colored by corresponding genotypes. The x axis shows the norovirus genomic region, while the height of each clone indicates the depth of coverage at each given genome position.

We found five mixed-infection cases in our virus collection, three from cross-sectional studies and two from cohort studies. The three cases from the cross-sectional study included mixed-infections with GII.14 and GII.4, GII.2 and GII.14, and GII.12 and GII.6 (Fig 10A). By applying a clonal population analysis, which determines the clones (haplotypes) present in the sample by association of NGS reads by similarity [62,63], we reconstructed the consensus sequences from multiple clones or genotypes at the near full-length level (>7400 nt, Fig 10A). Two cases from Peruvian birth cohort and family cohort studies included mixed-infection with GII.4 and GII.3, and GII.NA2 and GII.4 viruses. In these studies, stool samples were collected regularly, which allowed us to follow up virus diversification during the shedding phase of these two reinfection cases (Fig 10B and 10C). Child NV066X was infected with GII.4 at Day 0 (September 10, 2012), and norovirus positive stool samples were collected until Day 20. On Day 7, this child was infected with GII.3 virus, and both viruses were detected until Day 20. Nearly full-length consensus sequences were reconstructed from both viruses at each time point. Based on the average depth of coverage, the GII.3 virus predominated on Day 7 and 11, but GII.4 virus re-emerged as the predominant population on Day 16 and 20. This trend was confirmed by genotype-specific qPCR from extracted RNA samples (S10A Fig). The other child (PX127) was infected with GII.4 and GII.NA2 viruses on or before April 28, 2008 (Day 0), and norovirus sequences were obtained until Day 29 (Fig 10C). On Day 0, most of the genomic reads were mapped against GII.4 virus (86% in total reads), while GII.NA2 was detected as a very minor population (6%). This prevalence was reverted by Day 15, with GII.NA2 becoming predominant on Day 15 and 21. Finally, GII.4 virus disappeared and only GII.NA2 virus was detected on Day 29. Genome titers calculated by qPCR recapitulated this trend (S10A Fig). The presence of mucosal IgA was measured using the stool samples and norovirus virus-like particles (VLPs) as antigens. Surprisingly, norovirus genotype-specific IgA response was detected only against one of the infecting genotypes, the major clone at the end of the infection, and not the one first cleared (S10B Fig). Viral mutants (subclones) were detected in both major viruses at the end of shedding periods, possibly as a result from the mucosal response (Fig 10B and 10C).

Importantly, no chimeric sequences (i.e. indication of recombinant genomes) were detected in any of the five cases described above. The NGS reads were clearly mapped against two reference genomes, and none of them were mapped across the two different reference sequences (Fig 10). No recombinant genomes were detected during the prolonged shedding either, which account for up to 4 weeks of mixed infections. Chimeric reads were further searched for those prolonged shedding cases using artificial chimeric genomes as references -sequence regions from two reference genomes were artificially exchanged at the ORF1/2 junction- to enforce the mapping tool to detect chimeric reads with no positive results (S11 and S12 Figs). One single chimeric read was detected in the GII.3 and GII.4 mixed infection at Day 7 (S11 Fig), but was not reproduced after a second NGS run.

### Limited frequency of intra-genotype recombination in human populations

**Fig 11:**
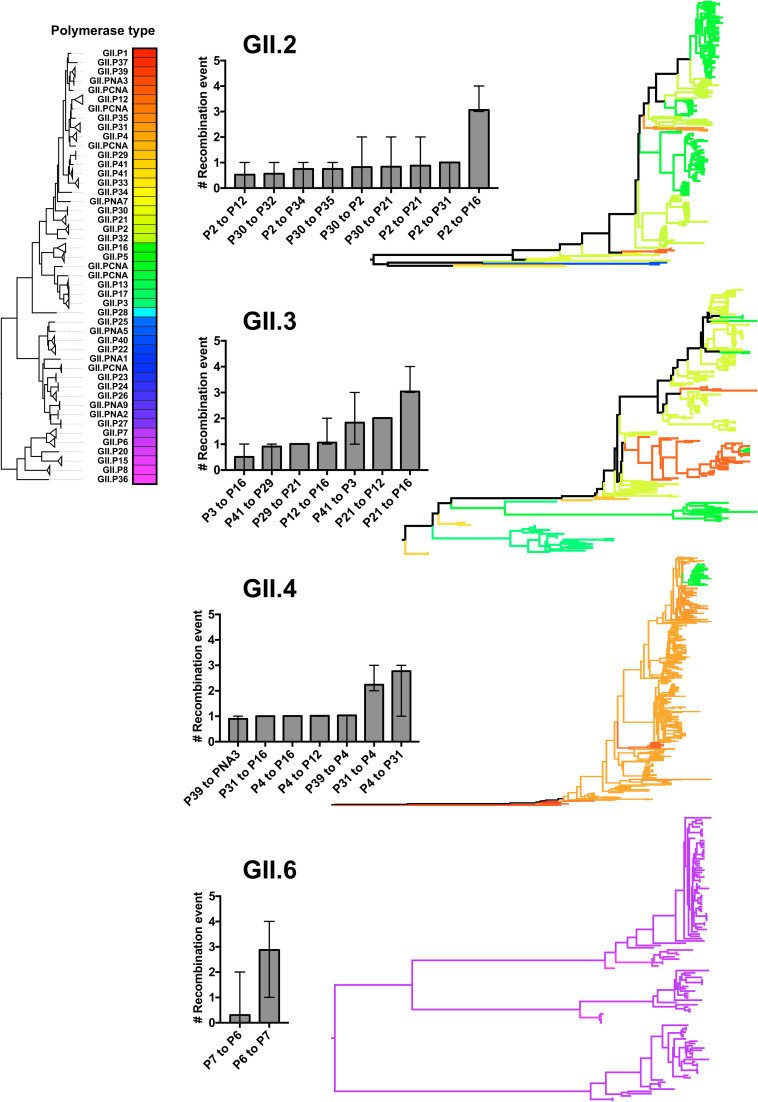
Limited number of recombination events in the evolutionary history of major norovirus genotypes. The maximum-clade credibility trees of GII.2, GII.3, GII.4, and GII.6 noroviruses were estimated and annotated with their corresponding polymerase types. Branches are color-coded by the polymerase types on the external tips and their ancestral nodes. The color for each polymerase type was determined from the phylogeny of the NS7-encoding nucleotide sequences. The direction and number of changes of polymerase types, i.e. recombination events, were estimated using Markov jump counting along the branches. Events with mean frequency >0.5 were summarized as bar graphs (the mean and the 95% highest posterior density interval).

Another evidence for the limited intra-genotype recombination of human noroviruses is revealed by tracking the changes in the polymerase type along the evolutionary trees of VP1 (Fig 11). Examination of the phylogenetic trees from four of the most frequent norovirus genotypes reconfirmed that most recombination events occurred with polymerase types from the same branches on the NS7 phylogenetic tree (Fig 11). The number of changes of polymerase types along branches, as counted with Markov-jump counting method [64], suggested limited recombination events during the evolution of these GII genotypes during the last 40 years. Thus, GII.2 viruses present two major polymerase types, GII.P2 and GII.P16, and recombination from GII.P2 to GII.P16 occurred three times throughout their evolution while recombination with other minor polymerase types occurred only once. In GII.6 viruses, recombination from GII.P6 to GII.P7, which are very close to each other, also occurred three times but not vice versa. The GII.4 viruses circulating in the 1970s and early 1980s presented the GII.P39 type, but by mid-1980s the polymerase type switched to GII.P4, which predominated until early 2010s. In 2012, the Sydney 2012 variant emerged as a recombinant presenting the GII.P31 type [50]. Recently, the GII.4 Sydney 2012 variant recombined to acquire a GII.P16 polymerase type, which is currently the dominant virus in multiple countries [32,65]. Recombination events including GII.P4 and GII.P31, which are genetically very similar to each other, occurred 2.2–2.8 times (mean; 95%HPD upper limit = 3) in Sydney 2012 and Osaka 2007 variants, while recombination to the GII.P16 occurred only once (Fig 11). No opposite direction of recombination (from GII.P16 to GII.P4 or GII.P31) was estimated from the tree. Finally, GII.3 viruses experienced multiple recombination events: three times from GII.P21 to GII.P16, two times from GII.P21 to GII.P12 and GII.P41 to GII.P3, and a single recombination event was recorded for GII.P3 to GII.P16, GII.P41 to GII.P29, GII.P29 to GI.P21, and GII.P12 to GII.P16. While GII.2 and GII.3 present over ten recombination events, that still could be regarded as a low number of recombination considering that this occurred over four decades. Together, this shows that recombination in human noroviruses is not a frequent event, or a small number of recombinant viruses are fit to emerge and circulate in the human population.

## Discussion

Because of the public health impact and development of new technologies to study virus genomics, the epidemiology and diversification of norovirus has been widely investigated [13,14,66]. Over the last two decades, a great number of norovirus sequences have been recorded in public databases; however, this public dataset is currently biased at the geographical and temporal level, and only limited genotypes and genomic regions have been widely sequenced. Thus, most records belong to capsid sequences from the major genotypes (GII.2, GII.3, GII.4, GII.6, and GII.17) detected in developed countries since the 2000s. In this study, we successfully closed some of the information gaps by adding new sequence information for 24 genotypes and over 80 noroviruses circulating prior to 2000. Removal of temporal bias improved the fitness of the molecular clock analyses and our estimates of norovirus evolution. Many studies have reported the evolutionary rate of noroviruses, with some suggesting that differences in the evolutionary rate among different norovirus genotypes could account for the emergence and predominance of certain viruses [21,67]. Our analyses showed that norovirus presented similar rates of evolution across the different ORFs and genotypes/variants (mean 1.37–5.38 × 10^−3^ substitutions/site/year) with overlapping 95%HPD intervals. Some genotypes, e.g., GII.1, GII.3, and GII.6, presented substitution rates that did not show overlapping of the 95%HPD intervals as compared with other genotypes; however, those differences were not related with the epidemic potential of the viruses. Thus, newly emerged or predominant viruses, like GII.2, GII.6, or GII.17 did not present higher rates of evolution as compared to other minor genotypes. Diversifying pressure on VP1, mostly on the P2 sub-domain, was detected in predominant genotypes and those with multiple variants. However, only viruses from the GII.4 genotype accumulate changes on the VP1 protein. Detection of diversifying pressure on non-GII.4 viruses with multiple variants suggests that these different lineages emerged, rapidly adapted to the human population, but showed low genetic robustness to accommodate major changes on their VP1 protein after decades of circulation and evolution. The significance of the co-circulation of multiple non-GII.4 variants is not completely understood. Changes in susceptibility and antigenicity have been attributed to the recent emergence of the new GII.17 variant that predominated during 2014–2015 in Asia [25,26]; however, additional studies are required to determine the phenotypic differences that could account for the emergence and co-circulation of the other non-GII.4 variants.

Similar to other viruses, emergence of new norovirus is probably marked by three evolutionary steps: (i) acquisition of mutations that would provide an initial advantage, (ii) subsequent mutations that would result in the proper adaptation to epidemic potential, and (iii) dispersion of the virus resulting in various mutations that follow a stochastic pattern [16,68]. These series of events have been shown to occur in the emergence of different GII.4 variants [14,16,69], but most recently in the emergence of predominant GII.17 viruses. With stronger surveillance systems, the emergence of GII.17 was characterized by the quick change from one transiently circulating variant (namely C, circulated during 2012–2015) to the predominant variant (namely D, which was detected since 2013 and is still reported in certain countries, e.g. Japan [70]). Thus, this rapid adaptation to the human population makes the detection of the intermediate viruses very difficult. In our historical samples, by focusing the attention on those less prevalent viruses, we detected multiple viruses that branched between defined variants or genotypes. While different hypotheses were presented on the emergence of novel norovirus (e.g. spill-over from hidden animal reservoirs [71] or variants originated in immunocompromised individuals [72]), it is possible that the genetic drift operates over long periods and the intermediate viruses are cryptically circulating in under-sampled populations, as shown by the recent detection of ‘novel’ noroviruses in active surveillance in communities [44,45,73] or country-wide monitoring systems [51]. Considering the cryptic genetic drift and rapid adaptation to achieve epidemic potential, we may need to accommodate multiple evolutionary steps, i.e. genetic drift, rapid adaptation, and exponential growth of viral population followed by predominance in the communities, to the evolutionary models or in-depth analyses of the intra-host evolution of noroviruses to fully understand the emergence of novel viruses.

A paradigm of norovirus epidemiology is that changes on the VP1 protein will prompt the emergence of new noroviruses by facilitating the escape from herd immunity [47]. While this seems to be the case for GII.4 noroviruses that presented continuous changes on the VP1, the recent predominance of GII.2 has been explained by changes on the viral polymerase [23]. Thus, it has been suggested that higher mutational rates, escape from T-cell immune responses to non-structural proteins, or increases in the replicative efficiency that results in higher levels of virus shedding could enhance transmissibility [33,35,69,74]. In that regard, the role of other non-structural proteins has been largely overlooked. Thus, in addition to mutations on the viral polymerase (NS7), our study found high variability and mutations on the NS1/2 (N-term) and NS4 (3A-like) proteins from epidemic vs. endemic GII.P16 and GII.P31 viruses. NS1/2 and NS4 proteins have been shown to induce replication complex formation by recruiting cellular membranes, and NS1/2 has been suggested to mediate and control the pathways of innate immunity [75,76]. Whether changes on these proteins are the consequence of founder effect or indeed enhance the epidemic potential from some viruses, warrants further research.

Another paradigm of norovirus epidemiology and evolution is the frequent interchange of genomic regions, by means of recombination, that results in new viruses with epidemic potential [12,27,28]. A hot spot for norovirus recombination has been identified at the ORF1/ORF2 overlapping region [27], reinforcing the role of non-structural proteins in the emergence of new noroviruses. Although a very large number of combinations of genotypes could be present in nature by means of recombination, our findings indicate that noroviruses show restriction in their ability to recombine with phylogenetically unrelated genotypes. This was supported by large-scale population genomics analyses, as well as the analysis of the intra-host diversity of patients with mixed infections. The latter provided a unique opportunity to determine the frequency of recombination among two viruses from the same phylogenetic cluster (i.e. GII.4[P31] and GII.3[P16]) that could result in epidemiologically relevant viruses, e.g. GII.4[P16]. While one chimeric read (0.005% of coverage) was detected from the second sample (day 7), this was not reproducible in a second experiment. These data indicate that recombination at the ORF1/2 overlapping regions may occur under strong restriction and less frequently than initially thought. Previous studies reported a small number of recombinant viruses with capsid and polymerase combinations that do not follow the proposed restriction in this study, e.g. GII.4[P7] or GII.7[P21] [28,30]. Careful interpretation should be given to those reports, as the recombinants were determined with short-length sequences that could be incorrectly typed and/or amplicons from separated regions of the genomes that could be an experimental artifact of mixed infections. Whether recombination events occur more frequently at the intra-genotype or at the variant level [41,50] remains to be determined. A limitation of our analyses is that it is technically difficult to define recombinants or false-positives among genetically similar viruses with intermediate viruses undetected.

Template switching between genomic and sub-genomic RNA is widely accepted as the molecular mechanism for ORF1/2 recombination in noroviruses [3,28]; however, there are no sufficient data to determine whether the sub-genomic or genomic RNA works as the acceptor molecule. Simmonds et al. predicted a stem-loop structure at the 3’ end of ORF1 (≥7 nt downstream of the (-) sub-genomic RNA start position) [60], that was shown to be part of the sub-genomic RNA promoter in murine norovirus [77]. If template switching occurs at the stem-loop as suggested in the copy-choice model [78], acceptor genome should be derived from genomic RNA with this stem-loop promoter sequence. Indeed, Bull et al. estimated the recombination breakpoints were located on average 16–19 nt upstream from the start of (+) ORF1/ORF2 overlap [29]. Our finding supports the idea that genomic RNA, not sub-genomic RNA, works as an acceptor template. The acceptor genomic RNA could be annealed with the donor template at the 3’ end of the ORF1, and thus the template-switching is restricted by the sequence identity on those regions between donor and acceptor RNA molecules. Based on these recombination restrictions, human noroviruses seem to be clustered into five recombination groups of viruses that do not genetically interact among each other. Restrictions on recombination of genes have been shown for multiple positive-sense RNA viruses, including enterovirus [79] and flavivirus [80]. Additionally, restrictions on the reassortment of genes have been reported for segmented viruses such as influenza virus and rotavirus [81,82]. These restrictions seem to be governed by protein-protein and protein-nucleic acid interactions [83–88]. This study supports the mechanism of restriction to nucleic acid-nucleic acid interactions during the processing of (-) RNA genomes. Additional studies are warranted to determine whether exchange of genomes among the different genotypes may provide fitness disadvantages [89–91], by which certain proteins interact better with the structural proteins or with host machinery during the replication process. Also, it is to be studied whether these restrictions occur in other positive-sense RNA viruses that utilize recombination as a mechanism of diversification [79,92].

Development of a simple norovirus genome sequencing protocol provided us the opportunity to analyze archival samples and different aspects of norovirus diversification and evolution. In conclusion, we showed that human noroviruses present (i) a similar rate of evolution at all genomic regions and genotype/variant levels, (ii) intermediate evolutionary states that might be a key for the adaptation to emerging viruses, (iii) mutations on non-structural proteins that could provide novel characteristics, and (iv) restrictions on the ability to recombine different (ORF1/ORF2) genomic regions, which results in co-circulating populations of viruses evolving independently. Moreover, the new sequence information and analyses reported here could provide baseline information for the study of future epidemics and ultimately the prevention of norovirus infection.

## Materials and methods

### Ethics statement

Studies were originally approved or exempted by IRB from each respective institution. Archival stool samples stored at different laboratories were analyzed anonymously and collectively exempted under FDA institutional IRB (16-069B).

### Archival samples

We retrospectively analyzed archived fecal samples positive for human norovirus, focusing on sequencing minor norovirus genotypes or historical viruses collected in the 1970s, 1980s, 1990s, and early 2000s (S1 Table). The samples were collected as part of studies conducted in nine laboratories [52,56,93–99] and the World Health Organization in different countries during 1976–1979 [100,101].

### Full-length RT-PCR and deep sequencing

Nearly full-length deep sequencing of archival viruses was retrospectively performed as previously described [14]. Briefly, complementary DNA (cDNA) was generated from the extracted viral RNA genome using the Maxima Minus First Strand cDNA Synthesis Kit (ThermoFisher Scientific) and a poly-A primer. The full-length viral genome amplification was done using the SequalPrep Long PCR Kit (Invitrogen) and primers listed in S3 Table. The resulting full-length viral genome amplicons (~7.5 kb) were run on the 1% agarose gel and extracted using Qiagen Gel Extraction Kit (Qiagen, California, USA). The gel-extracted amplicons were quantified using the Qubit dsDNA HS Assay Kit (ThermoFisher Scientific), and subjected to NGS using MiSeq system (illumina, California, USA). The library for NGS was prepared using the Nextera XT DNA Library Prep Kit (illumina), and the paired-end 2 × 250 bp sequence reads were obtained. Reads were quality-filtered (base quality score ≥ 20, and depth of coverage ≥ 10) and i) mapped against reference genome set (all genotypes and polymerase types implemented in Norovirus Genotyping Tool [102]) to screen the amplified genotypes, and ii) mapped against corresponding full- or nearly full-length reference genomes to reconstruct its consensus sequence using HIVE platform [62]. Samples were re-processed from the 10% stool suspension to confirm the mixed-infection if multiple genotypes were detected. Reads from mixed infection samples were then mapped against corresponding reference genomes followed by clonal population analysis using the Hexahedron Coordinated Clonal Analysis Tool implemented in the HIVE platform [63]. This clonal population analysis separately assembled all possible viral population (clones) contained in a single sample, and reconstructed their own consensus genomes at a near full-length level. Finally, chimeric reads were searched by HIVE using artificial chimeric genomes (400 nt ORF1/2 junction region) as references, and read-mapping against the references were visualized with MSAViewer [103]. Nearly-complete consensus genome sequences and raw NGS reads from shedding cases obtained in this study were deposited in GenBank (Accession numbers: MG706448, MK733201–MK733207, MW261787–MW261800, MW305481–MW305742; summarized in S1 Table) and SRA (BioProject accession number: PRJNA659534), respectively.

### Data summary

Along with our own genome set, we collected the nearly-full length (≥7000 nt) and/or VP1-encoding (≥1500 nt) GI, GII, GVIII, and GIX sequences from GenBank (as of March 30, 2020, S4 and S5 Tables). Sequences from animals, environment (e.g. sewage water), and immunocompromised patients were excluded. All the genomes were genotyped using Norovirus Genotyping Tool [102]. Collection years of the sequences were summarized using R v3.6.0 and GraphPad Prism v7. Geographic location (countries) were summarized and visualized using *maptools* and *mapplots* packages in R. Map shape file (1:10m Cultural Vector, Admin 0 –Countries) was obtained from Natural Earth website (https://www.naturalearthdata.com/downloads/10m-cultural-vectors/10m-admin-0-countries/, accessed on June 25, 2021).

### Sequence analysis

To avoid false phylogenetic signals arising from genome variability and/or recombination events, sequences were split into genogroups/genotypes and each genomic region in the encoding protein (NS1/2–7, VP1, and VP2). Sequences were then multiple-aligned using MUSCLE [104], MAFFT (for large dataset) [105], or TranslatorX (for protein-coding codon sequence alignment) [106]. Maximum-likelihood (ML) phylogenetic trees were built using PhyML for nucleotide and amino acid sequences, with best-fit substitution models estimated with Smart Model Selection and branch support estimated with approximate likelihood-ratio test implemented in PhyML [107]. Variants of each genotype were determined based on the cutoff of the 5% difference on the VP1 amino acid sequence and/or the proposed classification criteria using standard deviation of inter-/intra- variant patristic distance on VP1-based ML trees [11] using R and *ape* package.

### Evolutionary analysis of VP1 sequence

Evolutionary patterns, i.e. clock-likeness, rate of evolution, and selective pressure were analyzed with ORF2 (VP1-encoding) sequences. Clock-likeness of the dataset (genotypes/variants) was confirmed with linear regression analysis of root-to-tip distance using ML trees and TempEst v1.5 [108], with and without historical sequences. The rate of evolution (substitutions/site/year) was calculated using BEAST v1.10.4 with strict-clock model [109]. The SRD06 model was used for estimating the nucleotide substitution process of the VP1-encoding sequences. The population size was assumed to be constant throughout their evolutionary history. The first 10% of the logs from the Markov chain Monte Carlo runs were removed as a burn-in before summarizing the posterior values. Recombination frequency, i.e. change of polymerase type, throughout the evolutionary history was estimated using asymmetric Markov Jump counting method [64] implemented in BEAST v1.10.4. The amino acid accumulation over time (distance from the oldest virus within each genotype) was calculated using *phangorn* package and R, with JTT model as the best-fit amino acid substitution model (in most of the genotypes) estimated using Smart Model Selection. Site-by-site selective pressure on VP1 from each genotype was estimated using MEME (Mixed Effects Model of Evolution) that detects sites under episodic selection by assuming varying pressure across branches on the phylogenetic tree [110] and FUBAR (Fast, Unconstrained Bayesian AppRoximation) that assumes constant pressure on the entire tree [111], using Datamonkey (small dataset) [112] or HyPhy v2.5.14 (large dataset with ≥500 sequences) [113]. Statistically significant sites (P<0.05 with empirical Bayes Factor on internal branches>100 in MEME and Bayes Factor>0.9 in FUBAR) were plotted using GraphPad Prism 7. In order to reduce the sampling bias derived from recent large-amount sequence submissions of GII.2, GII.4, and GII.17 viruses, we generated subsampled dataset which includes a maximum of 20–30 randomly selected sequences per variant per year (n = 302 for GII.2, n = 821 for GII.4, and n = 204 for GII.17 viruses), and used for root-to-tip regression, evolutionary rate, and selective pressure analyses.

### Genome-wide analyses

To overview the evolutionary pattern of other proteins, the genome-wide nucleotide and amino acid sequence diversity was calculated as site-by-site Shannon entropy values using Entropy-One Tool (https://www.hiv.lanl.gov/content/sequence/ENTROPY/entropy_one.html). The amino acid accumulation pattern was also estimated for other nonstructural proteins and VP2 proteins from each genotype. Maximum 50 nt of the 5’ or 3’ end of the NS1/2 or VP2-encoding region, respectively, was removed from the analyses to exclude the sequence gaps derived from partial genomes. To account for the sampling bias by predominance of certain genotypes, the ML phylogenetic trees and Shannon entropy values for each genomic region was estimated using randomly subsampled dataset which included a maximum of two viruses from each polymerase and capsid genotype combination (n = 33 for GI and n = 119 for GII viruses). The evolutionary rate of ORF1 (NS1/2–NS7) and ORF3 (VP2) sequences were estimated using BEAST v1.10.4 as described above. The ORF1 sequences were partitioned by each non-structural protein and rate of evolution was jointly estimated. The amino acid distance from the oldest virus within each genotype was calculated for the NS4, NS7, and VP2 proteins using R as described above. To explore key amino acid residues on nonstructural and VP2 proteins that could be associated with global spread and the epidemic potential of noroviruses, phylogenetic analyses were conducted using the amino acid sequences from NS1/2–NS7 and VP2 from GII.P4, GII.P16, GII.P31, and GII.2 viruses. The ML phylogenetic trees were built using PhyML as indicated above, and ancestral sequences on the nodes that diverged into epidemic clusters were estimated using maximum likelihood method implemented in *phangorn* package in R. The amino acid mutations on the NS7 of epidemic viruses were mapped on the structural model of the polymerase from GII.P4 virus (PDB number 4QPX) using UCSF Chimera v 1.11. Recombination combination was tabulated using the nearly full-length sequence dataset with their polymerase and capsid genotype information, and visualized as a heatmap using GraphPad Prism v7.

### ORF1/2 junction analysis

The 400 nt sequence spanning the ORF1/2 junction region was extracted from the nearly full-length sequence dataset. The Shannon entropy values on this junction region were calculated using Entropy-One Tool as described above. In order to determine the restriction factors on recombination, we explored the 2D-MDS map generated from pairwise nucleotide differences on the junction region. The junction region was split into four non-overlapping windows (1–100, 101–200, 201–300, and 301–400 nt) and/or functional regions (3’ end ORF1, 5’ end sub-genomic region, predicted stem-loop, and linker sequence between predicted sub-genomic promoter and sub-genomic RNA [60]), and corresponding 2D-MDS maps were generated using R and *stat* package.

### Prolonged shedding of mix infections

We detected two mixed infection cases from birth cohort [93] and family cohort studies conducted in Lima, Peru. By following up those two cases, we successfully observed 20-days or 29-days prolonged shedding of mixed infection with two different genotypes: one child with GII.3 and GII.4, and another with GII.4 and GII.NA2, respectively. In addition to NGS and subsequent clonal population analysis, we performed genotype-specific qPCR to quantify the viral load of each genotype during the shedding. Briefly, viral RNA was quantified by duplex one-step real-time PCR with GII-specific primers and genotype-specific probes (S6 Table) using TaqMan Fast Virus 1-Step Master Mix kit (Applied Biosystems). The genotype-specific control plasmids were generated in house with corresponding partial viral genomes inserted into pCI vectors (Promega). In addition, with the 10% PBS suspension of the stool samples, we detected human IgA response with corresponding VLPs as antigens by ELISA. The VLPs were produced as described elsewhere using baculovirus expression system [8,16,114] with VP1-encoding sequences from GII.3 (Maizuru010524; accession number EF547399), GII.4 (RockvilleD1; KY424328), and GII.NA2 (PNV06929; MG706448) viruses.

## Supporting information

S1 Fig Limited diversifying selection on major capsid protein (VP1) from norovirus variants. Episodic diversifying selection was estimated for variants with ≥20 sequences using MEME (Mixed Effects Model of Evolution) method. Statistically significant positively selected sites (P<0.05 with empirical Bayes Factor on internal branches>100) were counted and summarized in a bar plot. Names of the GII.4 variants are abbreviated as follows; GR: Grimsby 1995, FH: Farmington Hills 2002, HT: Hunter 2004, SA: Sakai 2003, YE: Yerseke 2006a, DH: Den Haag 2006b, OS: Osaka 2007, AP: Apeldoorn 2007, NO: New Orleans 2009, SY: Sydney 2012.
(TIF)

S2 Fig Accumulation of amino acid mutations on NS4 proteins from human noroviruses. Amino acid distance was calculated from the oldest viruses for each given polymerase type from (A) GI and (B) GII viruses. Only polymerase types with data from samples with ≥5 sequences were analyzed. Lines represent the linear regression for amino acid mutations occurring during a given time span for each type.
(TIF)

S3 Fig Accumulation of amino acid mutations on NS7 proteins from human noroviruses. Amino acid distance was calculated from the oldest viruses for each given polymerase type from (A) GI and (B) GII viruses. Only polymerase types with data from samples with ≥5 sequences were analyzed. Lines represent the linear regression for amino acid mutations occurring during a given time span for each type.
(TIF)

S4 Fig Accumulation of amino acid mutations on VP2 proteins from human noroviruses. Amino acid distance was calculated from the oldest viruses for each given genotype from (A) GI, (B) GII, and (C) GIX viruses. Only genotypes with data from samples with ≥5 sequences were analyzed. Variants within each genotype were separately analyzed and are shown with different colors. Lines represent the linear regression for amino acid mutations occurring during a given time span for each genotype or variant.
(TIF)

S5 Fig Non-structural proteins from epidemic viruses differ from endemic viruses. (A) Maximum-likelihood phylogenetic analyses of non-structural proteins from epidemic viruses GII.P16 (top) and GII.P31 (bottom) indicated mutations on the epidemic clusters (colored by red) from the endemic viruses (colored by black). Mutations in bold indicate those as a single mutation and those in non-bold indicate there are other minor mutations detected at the population level. The values on the mutations show branch support provided by approximate likelihood-ratio test. (B) Amino acid mutations from epidemic viruses were mapped on the structural model of viral RNA polymerase (GII.P4; PDB number 4QPX). The incorporated RNA molecule is highlighted in yellow in the front view.
(TIF)

S6 Fig Phylogenetic analyses of non-structural proteins from GII.P4 noroviruses. Maximum-likelihood phylogenetic trees of non-structural proteins from epidemic and endemic GII.P4 viruses. Epidemic viruses were color-coded by variant. Endemic viruses are indicated by black circles.
(TIF)

S7 Fig Phylogenetic trees of VP2 proteins from GII.2 and GII.4 noroviruses. Maximum-likelihood phylogenetic trees of VP2 proteins from (A) epidemic GII.2 and (B) epidemic GII.4 viruses. The values on the mutations show branch support provided by approximate likelihood-ratio test. Endemic viruses were represented by black circles, and epidemic viruses were color-coded as indicated in the legend.
(TIF)

S8 Fig Genome-wide analyses confirm restrictions on ORF1/ORF2 recombination. Viruses were grouped phylogenetically and the number of viruses with a given capsid and polymerase types was recorded in each cell. Phylogenetic trees were calculated using all the non-structural and capsid proteins from a subsampled dataset: a maximum of two viruses from each combination of genotype and polymerase type. The colored boxes in the matrix indicate the recombination groups associated with the phylogenetic clustering on the NS7-encoding nucleotide sequences as defined in Fig 8.
(TIF)

S9 Fig Multidimensional Scaling Analysis on the nucleotide sequence variation at the ORF1/2 junction region. Multidimensional Scaling Analysis (MDS) of the noroviruses based on the nucleotide diversity at ORF1/2 junction region. Viruses are colored based on their recombination group as defined in Fig 8. We focused on predominant viruses from different recombination groups that presented multiple genotypes/polymerase types (i.e. GII.P7, GII.P16, GII.2, GII.3, and GII.4 viruses), which were highlighted with black in the MDS maps.
(TIF)

S10 Fig Virus genome titer and IgA response during mixed infection and prolonged shedding of two different norovirus genotypes. (A) Virus genome copies from two mixed-infection cases were quantified using genotype-specific qPCR system. Day 0 was set to the first day of norovirus positive, and virus titer was quantified up to 29 days post-infection. Limit of quantification in the qPCR was indicated by dashed line. (B) IgA titer in stool samples was measured by ELISA using VLPs of infected norovirus genotypes as antigens. The y-axis indicates OD values quantified at 405 nm. The average OD value from negative controls was indicated by dashed line. In both (A) and (B), left graphs present results from a cohort child NV066X and right show those from PX127.
(TIF)

S11 Fig NGS data from shedding samples from a child (NV066X) infected with GII.3 and GII.4 noroviruses. The 250 nt short reads from full-length PCR amplicons from cohort child NV066X were mapped against artificial chimeric reference genomes (GII.3/GII.4 or GII.4/GII.3) to explore the evidence of any mosaic genomes (i.e. recombinants) generated during the shedding phase from the individual infected with GII.3 and GII.4 noroviruses. The chimeric reference genomes were generated by switching the ORF1 and ORF2 regions between the GII.3 and GII.4 sequences.
(TIF)

S12 Fig NGS data from shedding samples from a child (PX127) infected with GII.4 and GII.NA2 noroviruses. The 250 nt short reads from full-length PCR amplicons from cohort child PX127 were mapped against artificial chimeric reference genomes (GII.4/GII.NA2 or GII.NA2/GII.4) to explore the evidence of any mosaic genomes (i.e. recombinants) generated during the shedding phase from the individual infected with GII.4 and GII.NA2 noroviruses. The chimeric reference genomes were provided by switching the ORF1 and ORF2 regions between the GII.4 and GII.NA2 sequences.
(TIF)

S1 Table Archival samples sequenced in this study. (XLSX)

S2 Table Evolutionary parameters and estimates of the VP1-encoding sequences. (XLSX)

S3 Table Primers utilized for full-length genome amplification. (XLSX)

S4 Table Nearly-full length norovirus sequences downloaded from GenBank in this study. (XLSX)

S5 Table Norovirus VP1 sequences downloaded from GenBank in this study. (XLSX)

S6 Table Primers and probes utilized for genomic quantification. (XLSX)
