# Supplementary figures and images for: Genome-wide analyses of human noroviruses provide insights on evolutionary dynamics and evidence of coexisting viral populations evolving under recombination constraints
Source: PLoS Pathog. 2021 Jul 13;17(7):e1009744. doi: 10.1371/journal.ppat.1009744 (PMC8318288; doi:10.1371/journal.ppat.1009744)

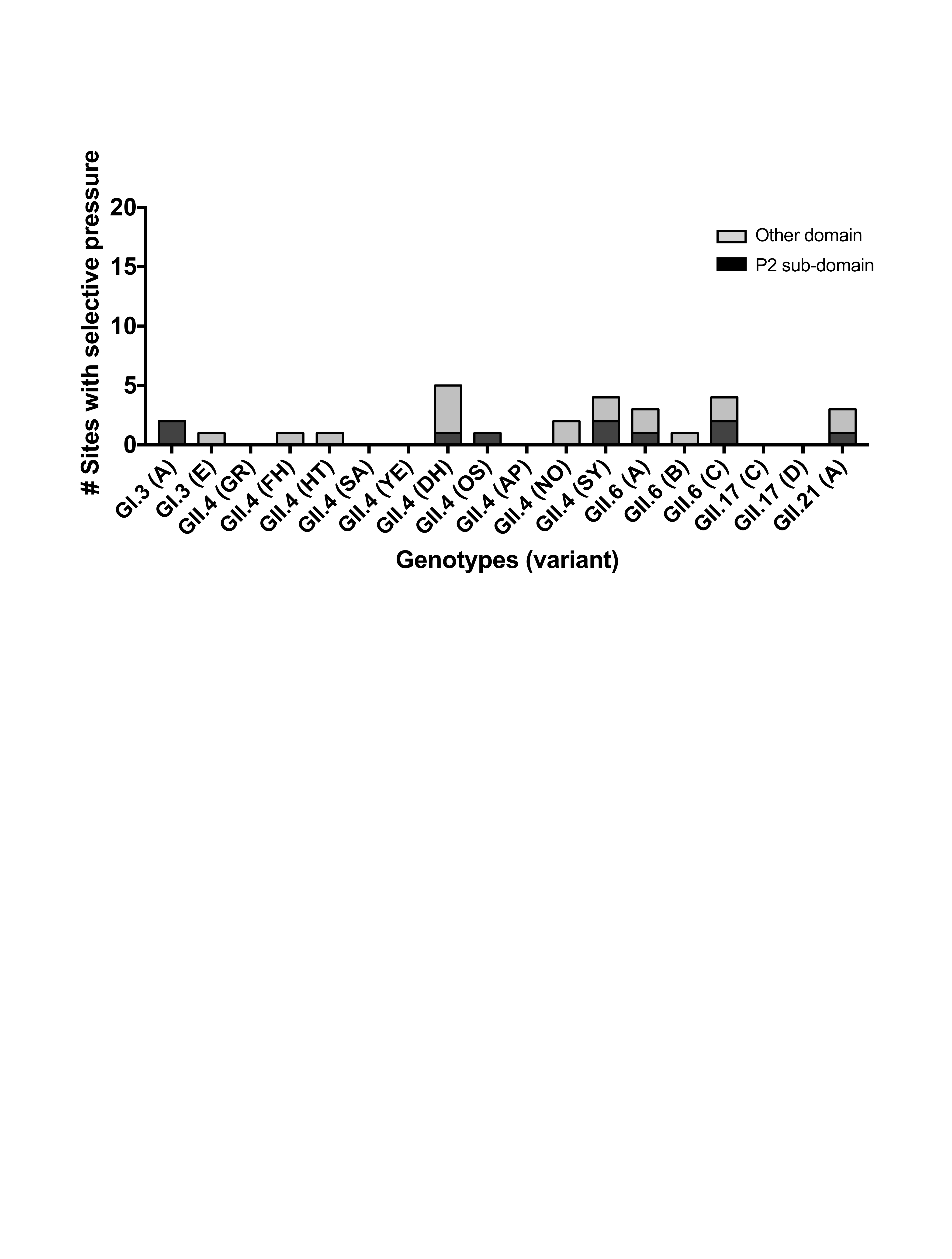

Supplement: S1 Fig — Episodic diversifying selection was estimated for variants with ≥20 sequences using MEME (Mixed Effects Model of Evolution) method. Statistically significant positively selected sites (P<0.05 with empirical Bayes Factor on internal branches>100) were counted and summarized in a bar plot. Names of the GII.4 variants are abbreviated as follows; GR: Grimsby 1995, FH: Farmington Hills 2002, HT: Hunter 2004, SA: Sakai 2003, YE: Yerseke 2006a, DH: Den Haag 2006b, OS: Osaka 2007, AP: Apeldoorn 2007, NO: New Orleans 2009, SY: Sydney 2012. (TIF) [file ppat.1009744.s001.tif]

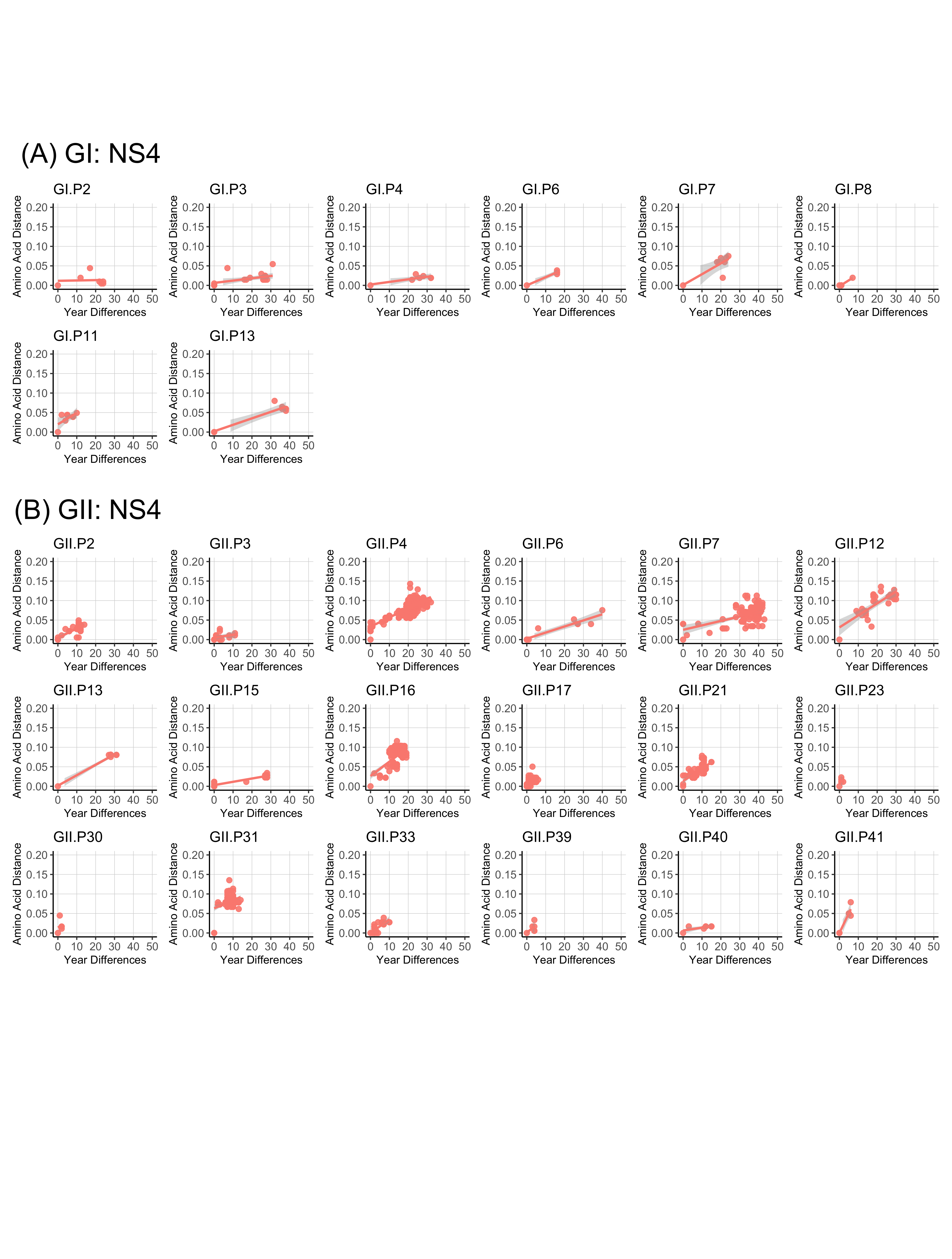

Supplement: S2 Fig — Amino acid distance was calculated from the oldest viruses for each given polymerase type from (A) GI and (B) GII viruses. Only polymerase types with data from samples with ≥5 sequences were analyzed. Lines represent the linear regression for amino acid mutations occurring during a given time span for each type. (TIF) [file ppat.1009744.s002.tif]

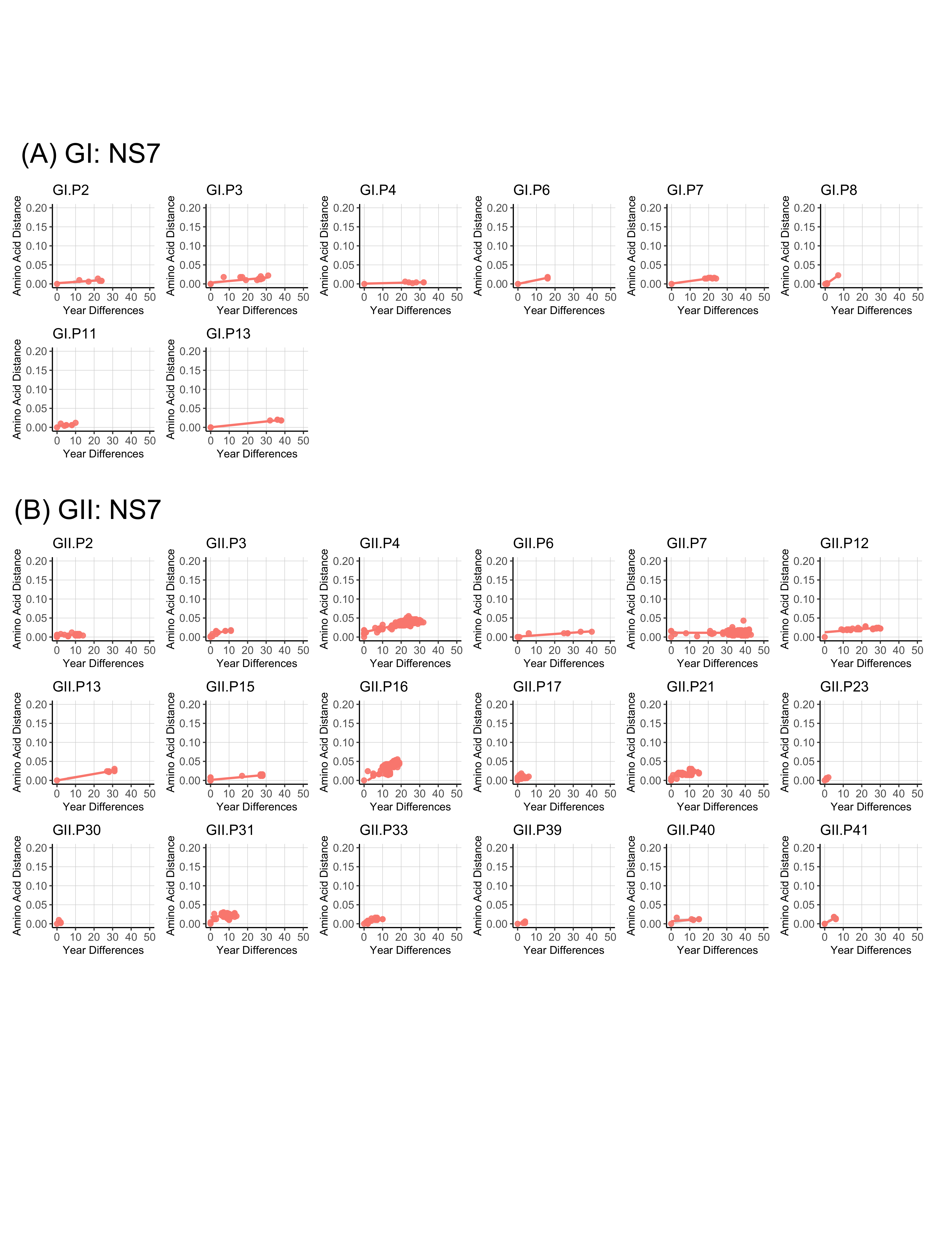

Supplement: S3 Fig — Amino acid distance was calculated from the oldest viruses for each given polymerase type from (A) GI and (B) GII viruses. Only polymerase types with data from samples with ≥5 sequences were analyzed. Lines represent the linear regression for amino acid mutations occurring during a given time span for each type. (TIF) [file ppat.1009744.s003.tif]

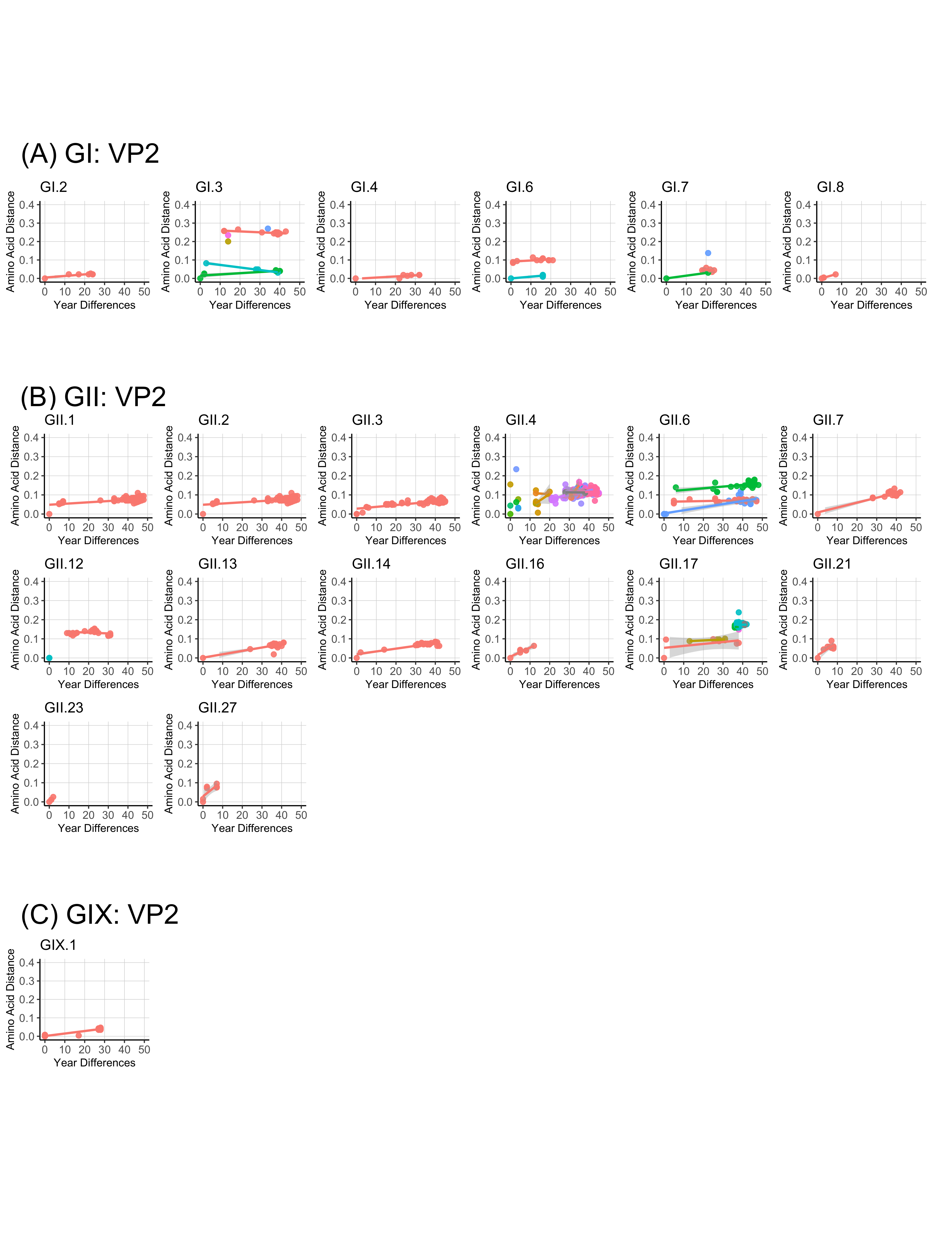

Supplement: S4 Fig — Amino acid distance was calculated from the oldest viruses for each given genotype from (A) GI, (B) GII, and (C) GIX viruses. Only genotypes with data from samples with ≥5 sequences were analyzed. Variants within each genotype were separately analyzed and are shown with different colors. Lines represent the linear regression for amino acid mutations occurring during a given time span for each genotype or variant. (TIF) [file ppat.1009744.s004.tif]

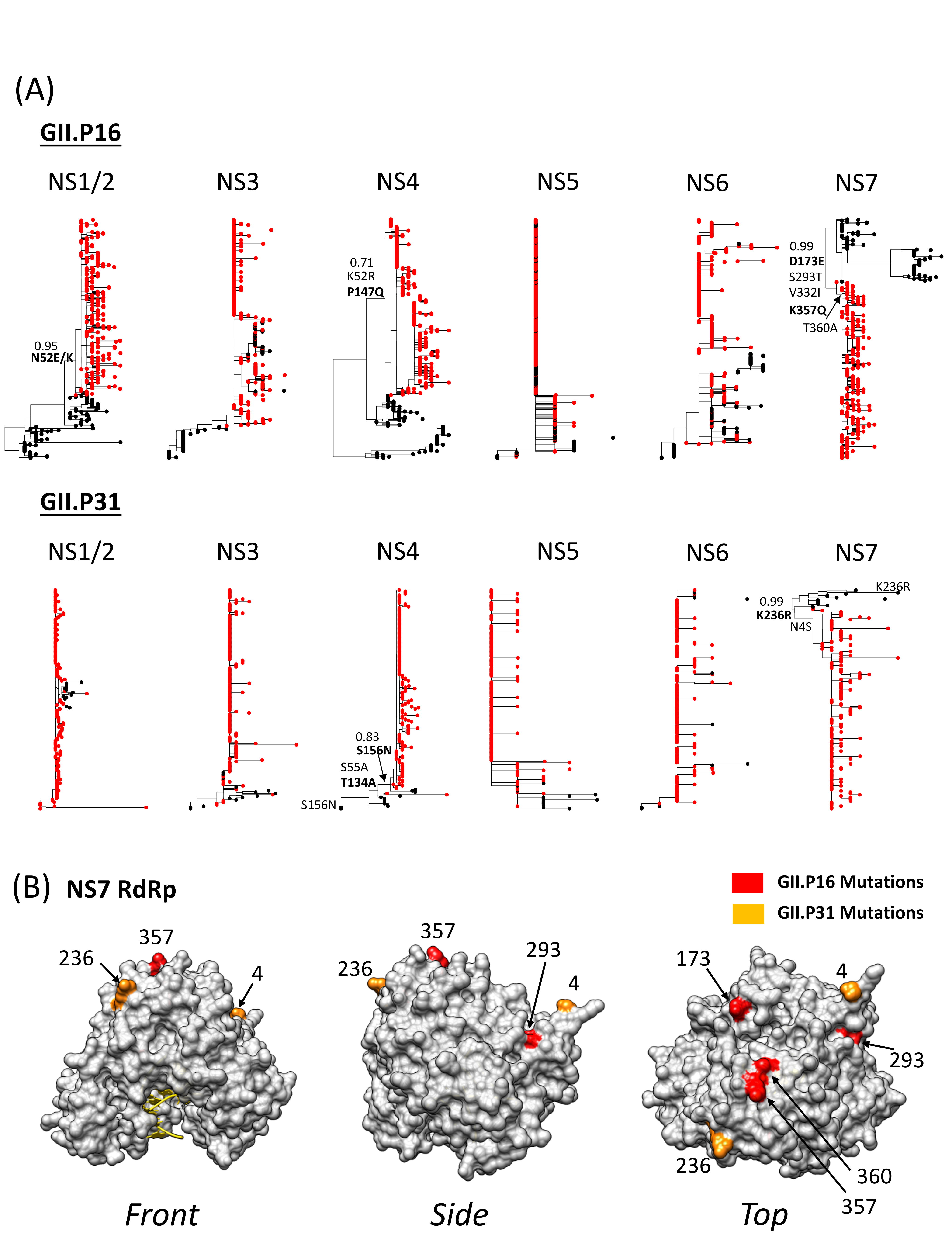

Supplement: S5 Fig — (A) Maximum-likelihood phylogenetic analyses of non-structural proteins from epidemic viruses GII.P16 (top) and GII.P31 (bottom) indicated mutations on the epidemic clusters (colored by red) from the endemic viruses (colored by black). Mutations in bold indicate those as a single mutation and those in non-bold indicate there are other minor mutations detected at the population level. The values on the mutations show branch support provided by approximate likelihood-ratio test. (B) Amino acid mutations from epidemic viruses were mapped on the structural model of viral RNA polymerase (GII.P4; PDB number 4QPX). The incorporated RNA molecule is highlighted in yellow in the front view. (TIF) [file ppat.1009744.s005.tif]

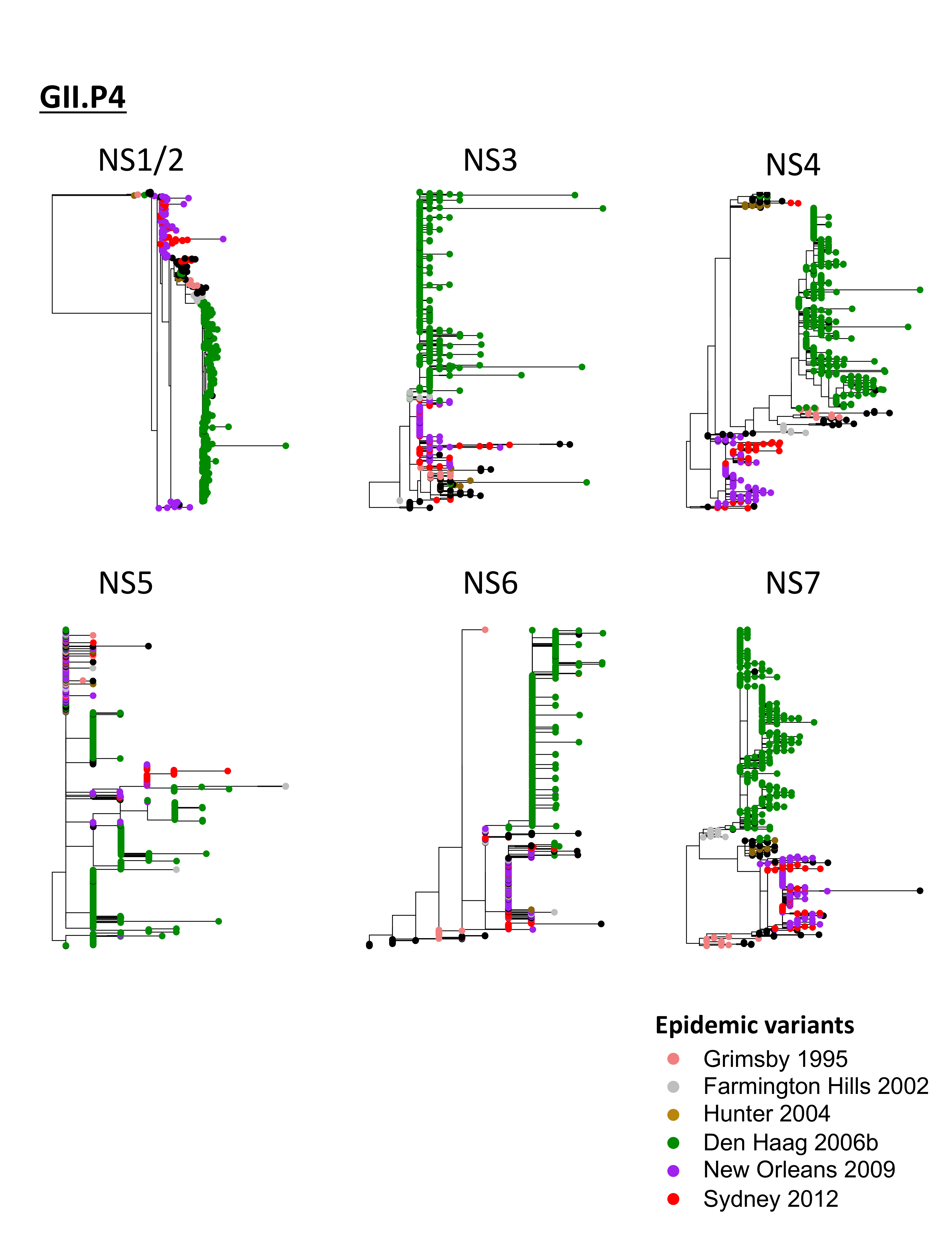

Supplement: S6 Fig — Maximum-likelihood phylogenetic trees of non-structural proteins from epidemic and endemic GII.P4 viruses. Epidemic viruses were color-coded by variant. Endemic viruses are indicated by black circles. (TIF) [file ppat.1009744.s006.tif]

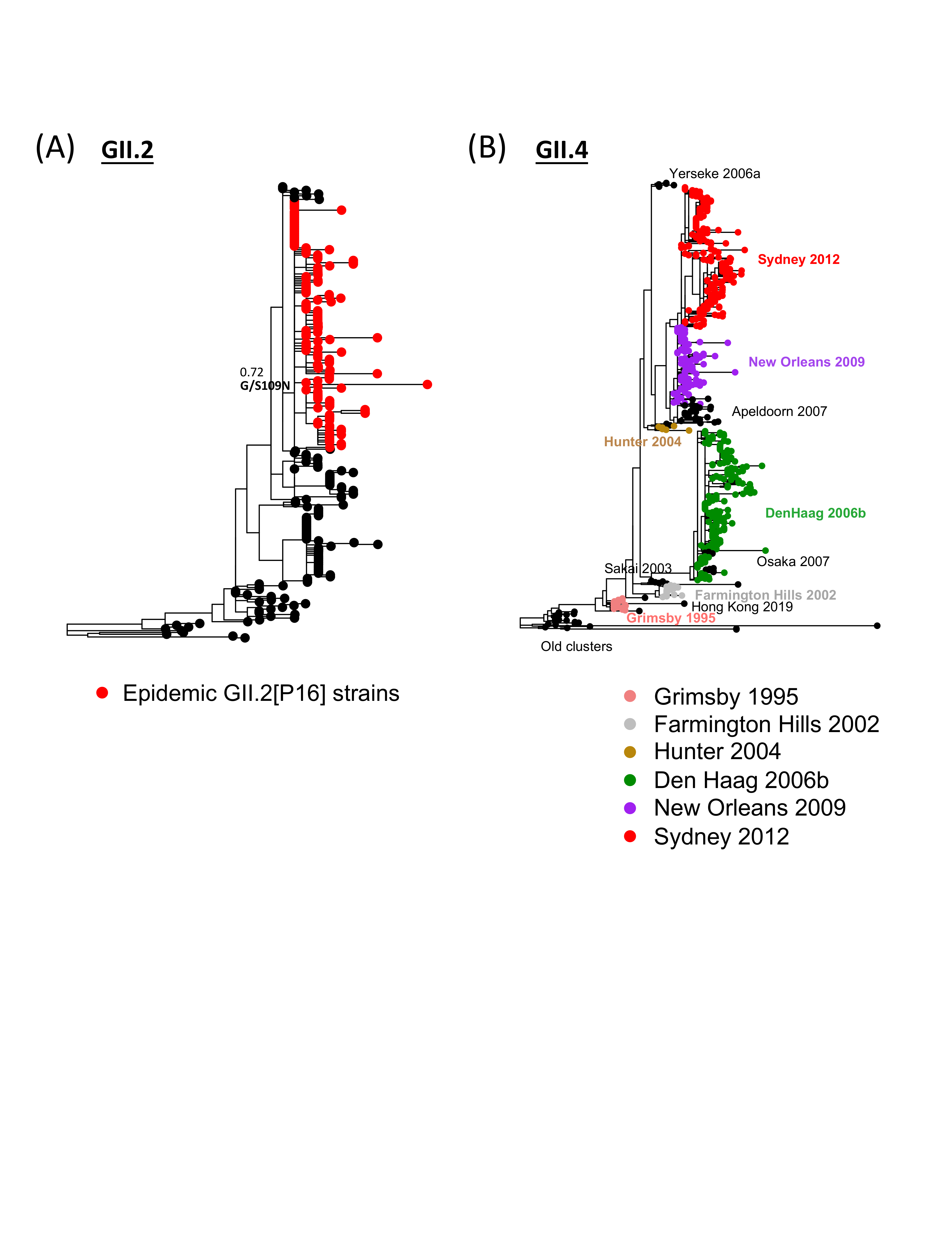

Supplement: S7 Fig — Maximum-likelihood phylogenetic trees of VP2 proteins from (A) epidemic GII.2 and (B) epidemic GII.4 viruses. The values on the mutations show branch support provided by approximate likelihood-ratio test. Endemic viruses were represented by black circles, and epidemic viruses were color-coded as indicated in the legend. (TIF) [file ppat.1009744.s007.tif]

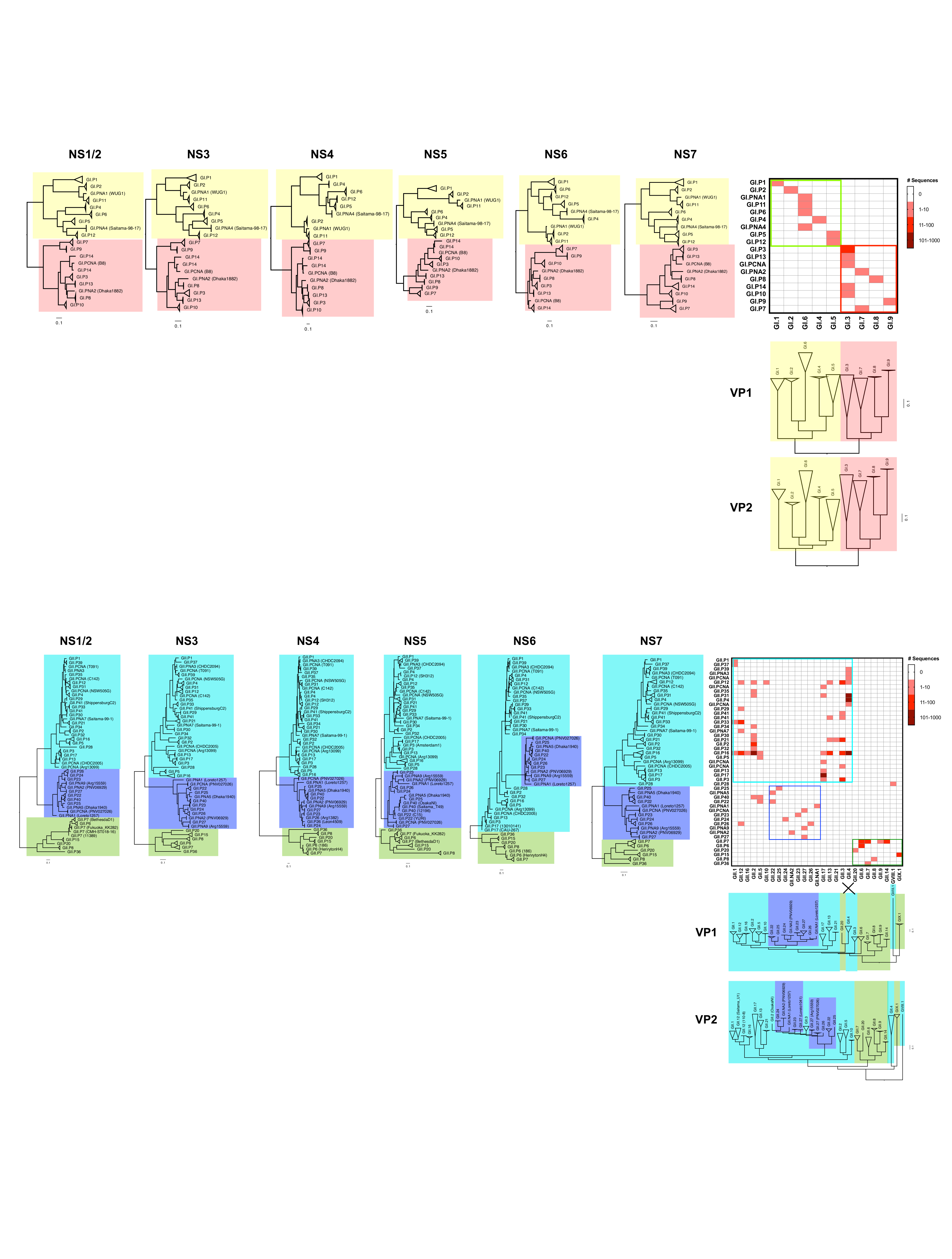

Supplement: S8 Fig — Viruses were grouped phylogenetically and the number of viruses with a given capsid and polymerase types was recorded in each cell. Phylogenetic trees were calculated using all the non-structural and capsid proteins from a subsampled dataset: a maximum of two viruses from each combination of genotype and polymerase type. The colored boxes in the matrix indicate the recombination groups associated with the phylogenetic clustering on the NS7-encoding nucleotide sequences as defined in Fig 8. (TIF) [file ppat.1009744.s008.tif]

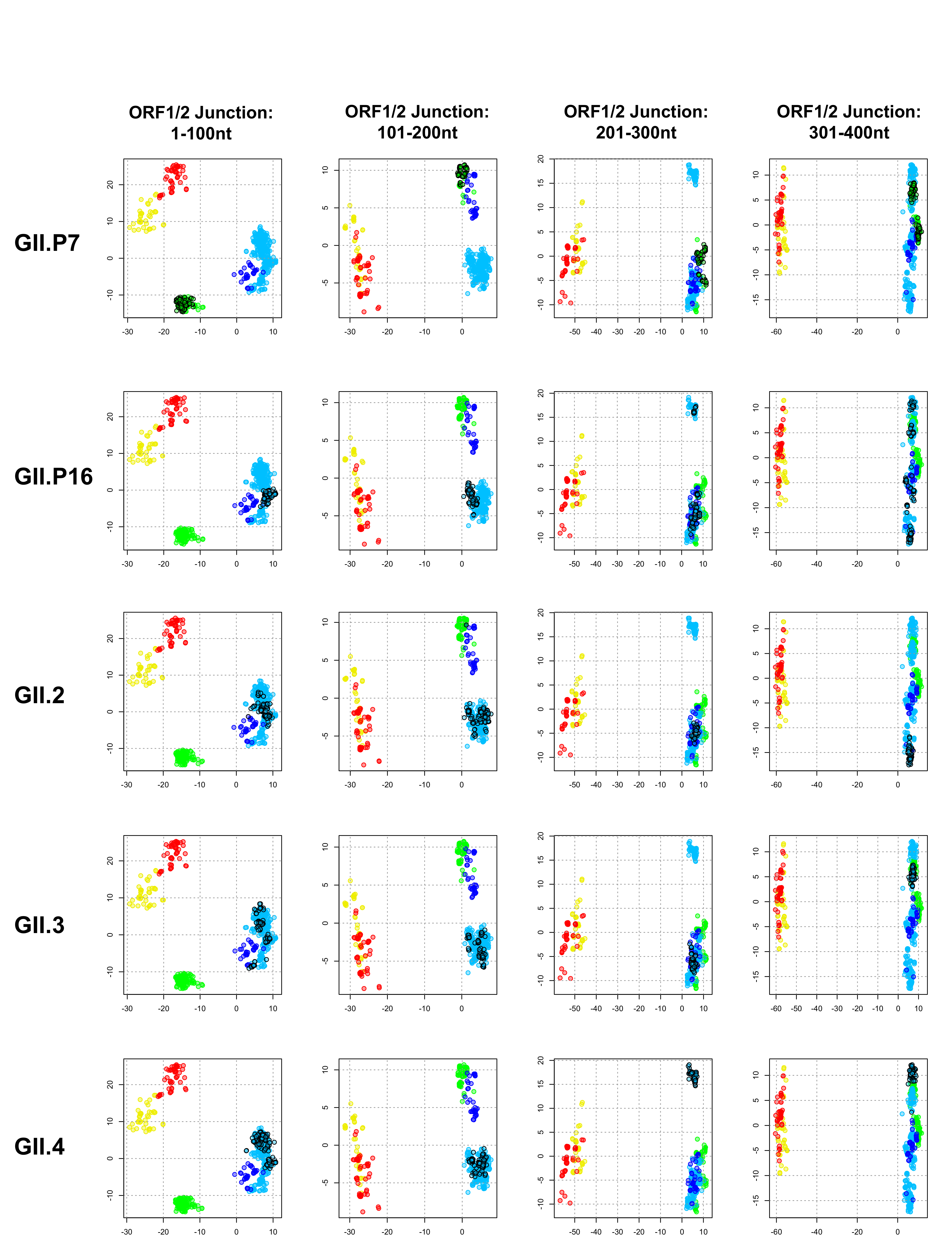

Supplement: S9 Fig — Multidimensional Scaling Analysis (MDS) of the noroviruses based on the nucleotide diversity at ORF1/2 junction region. Viruses are colored based on their recombination group as defined in Fig 8. We focused on predominant viruses from different recombination groups that presented multiple genotypes/polymerase types (i.e. GII.P7, GII.P16, GII.2, GII.3, and GII.4 viruses), which were highlighted with black in the MDS maps. (TIF) [file ppat.1009744.s009.tif]

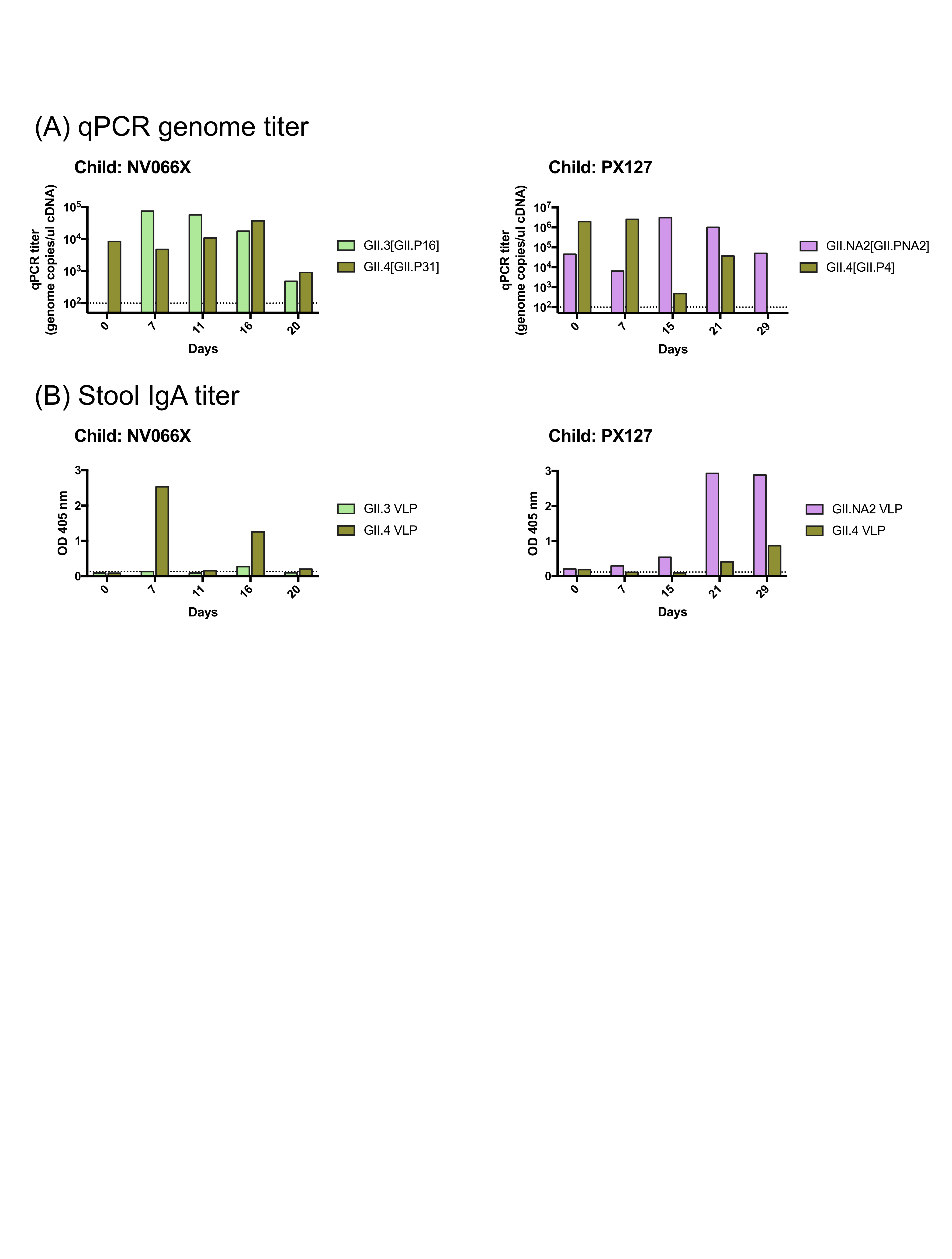

Supplement: S10 Fig — (A) Virus genome copies from two mixed-infection cases were quantified using genotype-specific qPCR system. Day 0 was set to the first day of norovirus positive, and virus titer was quantified up to 29 days post-infection. Limit of quantification in the qPCR was indicated by dashed line. (B) IgA titer in stool samples was measured by ELISA using VLPs of infected norovirus genotypes as antigens. The y-axis indicates OD values quantified at 405 nm. The average OD value from negative controls was indicated by dashed line. In both (A) and (B), left graphs present results from a cohort child NV066X and right show those from PX127. (TIF) [file ppat.1009744.s010.tif]

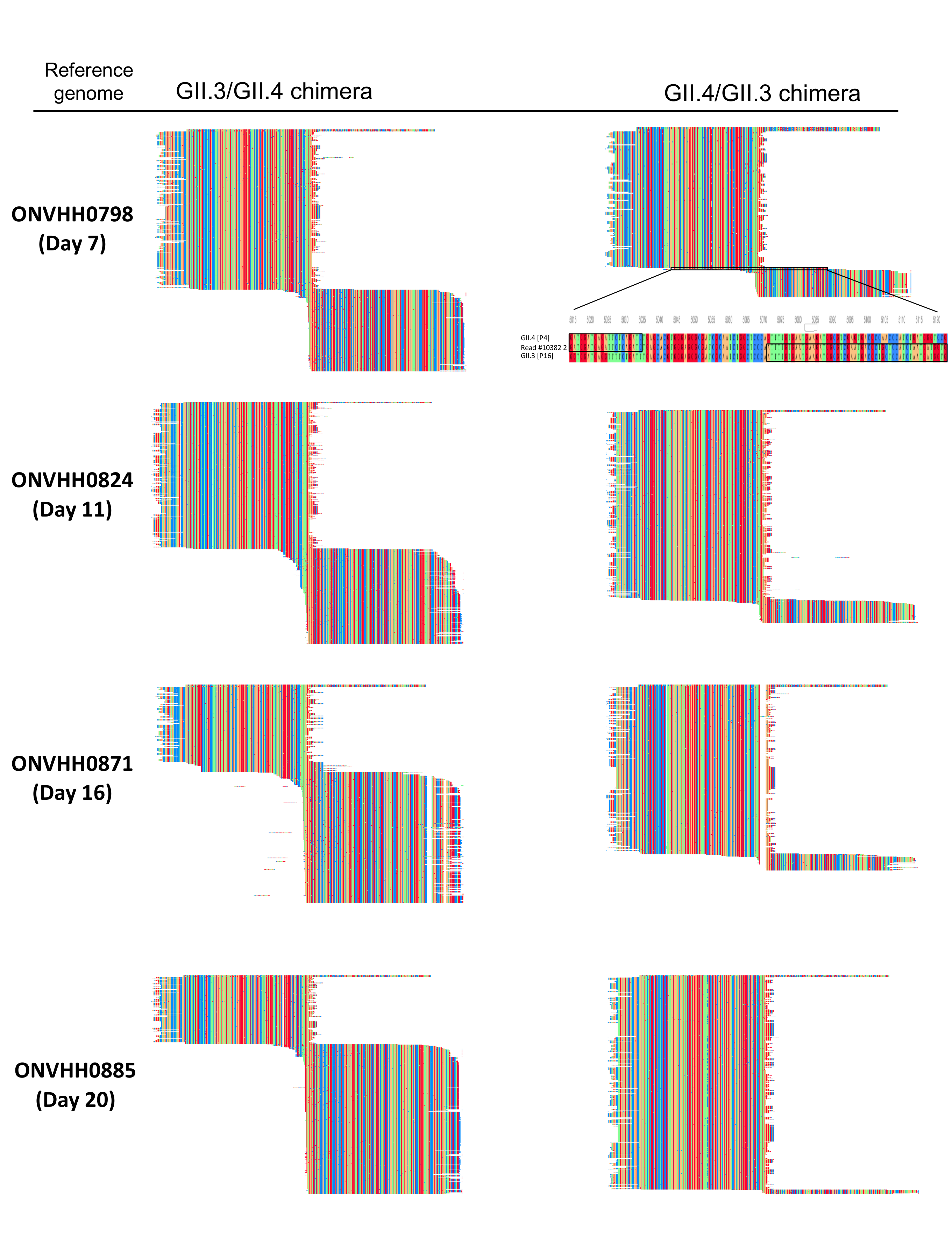

Supplement: S11 Fig — The 250 nt short reads from full-length PCR amplicons from cohort child NV066X were mapped against artificial chimeric reference genomes (GII.3/GII.4 or GII.4/GII.3) to explore the evidence of any mosaic genomes (i.e. recombinants) generated during the shedding phase from the individual infected with GII.3 and GII.4 noroviruses. The chimeric reference genomes were generated by switching the ORF1 and ORF2 regions between the GII.3 and GII.4 sequences. (TIF) [file ppat.1009744.s011.tif]

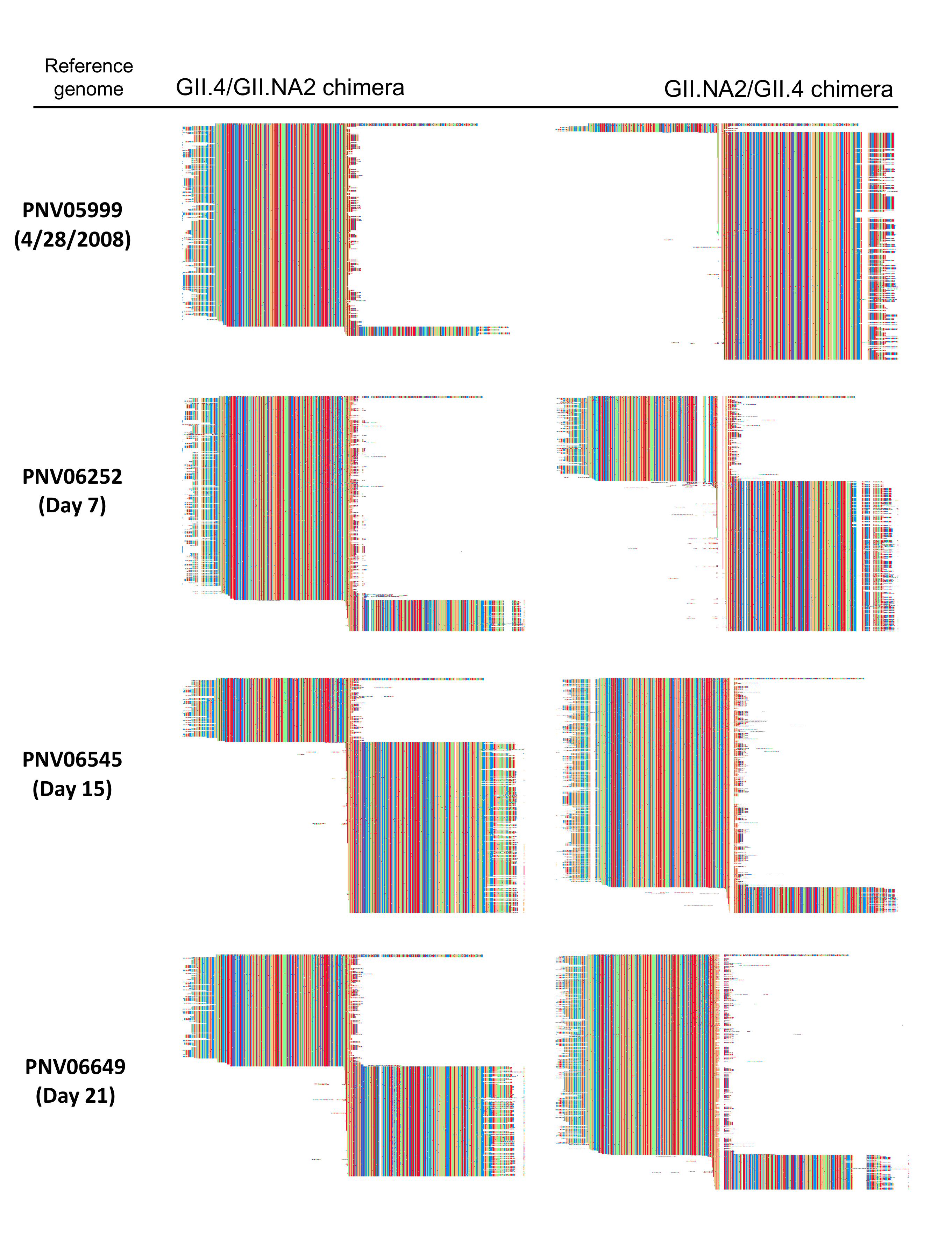

Supplement: S12 Fig — The 250 nt short reads from full-length PCR amplicons from cohort child PX127 were mapped against artificial chimeric reference genomes (GII.4/GII.NA2 or GII.NA2/GII.4) to explore the evidence of any mosaic genomes (i.e. recombinants) generated during the shedding phase from the individual infected with GII.4 and GII.NA2 noroviruses. The chimeric reference genomes were provided by switching the ORF1 and ORF2 regions between the GII.4 and GII.NA2 sequences. (TIF) [file ppat.1009744.s012.tif]
